# Examining the Impact of an mHealth Behavior Change Intervention With a Brief In-Person Component for Cancer Survivors With Overweight or Obesity: Randomized Controlled Trial

**DOI:** 10.2196/24915

**Published:** 2021-07-05

**Authors:** Jane C Walsh, Janice Richmond, Jenny Mc Sharry, AnnMarie Groarke, Liam Glynn, Mary Grace Kelly, Owen Harney, Jenny M Groarke

**Affiliations:** 1 School of Psychology National University of Ireland, Galway Galway Ireland; 2 Letterkenny University Hospital Donegal Ireland; 3 Health Research Institute and Graduate Entry Medical School University of Limerick Limerick Ireland; 4 Centre for Improving Health-Related Quality of Life School of Psychology Queen's University Belfast Belfast United Kingdom

**Keywords:** cancer survivors, overweight, obesity, health behavior, goals, accelerometry, text messaging, technology, Ireland, self-management, mobile phone

## Abstract

**Background:**

Cancer survivorship in Ireland is increasing in both frequency and longevity. However, a significant proportion of cancer survivors do not reach the recommended physical activity levels and have overweight. This has implications for both physical and psychological health, including an increased risk of subsequent and secondary cancers. Mobile health (mHealth) interventions demonstrate potential for positive health behavior change, but there is little evidence for the efficacy of mobile technology in improving health outcomes in cancer survivors with overweight or obesity.

**Objective:**

This study aims to investigate whether a personalized mHealth behavior change intervention improves physical and psychological health outcomes in cancer survivors with overweight or obesity.

**Methods:**

A sample of 123 cancer survivors (BMI≥25 kg/m^2^) was randomly assigned to the standard care control (n=61) or intervention (n=62) condition. Group allocation was unblinded. The intervention group attended a 4-hour tailored lifestyle education and information session with physiotherapists, a dietician, and a clinical psychologist to support self-management of health behavior. Over the following 12 weeks, participants engaged in personalized goal setting to incrementally increase physical activity (with feedback and review of goals through SMS text messaging contact with the research team). Direct measures of physical activity were collected using a Fitbit accelerometer. Data on anthropometric, functional exercise capacity, dietary behavior, and psychological measures were collected at face-to-face assessments in a single hospital site at baseline (T0), 12 weeks (T1; intervention end), and 24 weeks (T2; follow-up).

**Results:**

The rate of attrition was 21% (13/61) for the control condition and 14% (9/62) for the intervention condition. Using intent-to-treat analysis, significant reductions in BMI (*F*_2,242_=4.149; *P*=.02; *ηp^2^*=0.033) and waist circumference (*F*_2,242_=3.342; *P*=.04; *ηp^2^*=0.027) were observed in the intervention group. Over the 24-week study, BMI was reduced by 0.52 in the intervention condition, relative to a nonsignificant reduction of 0.11 in the control arm. Waist circumference was reduced by 3.02 cm in the intervention condition relative to 1.82 cm in the control condition. Physical activity level was significantly higher in the intervention group on 8 of the 12 weeks of the intervention phase and on 5 of the 12 weeks of the follow-up period, accounting for up to 2500 additional steps per day (mean 2032, SD 270).

**Conclusions:**

The results demonstrate that for cancer survivors with a BMI≥25 kg/m^2^, lifestyle education and personalized goal setting using mobile technology can yield significant changes in clinically relevant health indicators. Further research is needed to elucidate the mechanisms of behavior change and explore the capacity for mHealth interventions to improve broader health and well-being outcomes in the growing population of cancer survivors.

**Trial Registration:**

ISRCTN Registry ISRCTN18676721; https://www.isrctn.com/ISRCTN18676721

**International Registered Report Identifier (IRRID):**

RR2-10.2196/13214

## Introduction

### Background

There is an average of 35,000 new cases of cancer diagnosed each year in Ireland, representing a doubling of cases in the past 25 years [1]. At the same time, cancer survivorship in Ireland is also increasing, with survival at 5 years from diagnosis having increased from 42% in 1994 to 62% in 2019, with cancer survivors now making up 4% of the Irish population [1].

There is consistent evidence of a positive association between overweight, obesity, and all-cause morbidity and mortality [2]. A high BMI, poor diet, and lack of physical activity are identifiable risk factors for cancer development, and in cancer survivors, these factors can increase the risk of a secondary cancer or a subsequent primary cancer [3,4]. Cancer and its treatment can result in fatigue, physical inactivity, and loss of muscular strength [5]. Approximately 50% of cancer survivors have overweight or obesity [6], and research has linked obesity to a 46% increased risk of developing distant metastases in women [7]. Considering the consequences of morbidity and mortality, there is a need to facilitate rehabilitation of cancer survivors to reduce BMI, improve diet, and increase physical activity.

Health behavior change interventions can improve physical health outcomes, such as weight and BMI, as well as health behavior (eg, physical activity) and psychological health (eg, quality of life and well-being) in both the general population [8] and among cancer survivors [9,10]. The use of mobile technology (eg, apps and wearables) has been associated with significant reductions in weight and BMI [11] and significant increases in physical activity [12,13]. Mobile health (mHealth) interventions may be able to meet the need for cost-effective health behavior change interventions that can be incorporated into oncology services. Although mHealth interventions hold significant potential, adopting a theory- and evidence-based approach to intervention design is critical [14]. The behavior change wheel is a synthesis of 19 frameworks of behavior change [15]. The behavior change wheel, together with the behavior change technique (BCT) taxonomy, a standardized list of the active ingredients of behavior change interventions [16], enables researchers to develop and describe complex interventions in a systematic and rigorous manner.

Systematic review evidence suggests that the use of relevant BCTs significantly increased the success of weight loss programs [17]. A systematic review of existing healthy eating and physical activity interventions identified the BCTs *self-monitoring* in combination with *goal setting* and *feedback* as the most effective [18]. Furthermore, a meta-analysis of 30 randomized controlled trials (RCTs) that focused on increasing physical activity among cancer survivors reported that the BCTs *prompts*, *social rewards*, and *graded tasks* were associated with larger increases in physical activity in this population [19]. Consequently, these BCTs should be considered for inclusion in interventions aimed at increasing healthy eating and physical activity behaviors among cancer survivors.

Studies have found that both mHealth tools and relevant BCTs can lead to positive health behavior changes and weight loss; therefore, the delivery of BCTs through mHealth tools may be particularly effective. Digital interventions that included a greater number of BCTs were found to have larger effects on health behavior change than interventions with fewer BCTs [20]. A review and meta-analysis of studies using activity monitors found that in people with obesity, physical activity increases were greatest when the BCTs *goal setting* and *feedback* were incorporated in the mHealth intervention [21]. A systematic content analysis of the BCTs provided by wearable activity monitors concluded that most activity monitors included *self-monitoring*, *goal setting*, and *feedback* [22]. Incidentally, a review by Michie et al [18] found these to be the most effective BCTs for promoting healthy diet and physical activity overall.

mHealth interventions incorporating relevant BCTs have the potential to improve health and well-being outcomes. However, there are a limited number of mHealth interventions for cancer survivors that describe content in terms of BCTs. A recent systematic review identified 15 digital health behavior change interventions for cancer survivors, concluding that digital interventions may improve physical activity and reduce BMI; however, findings regarding dietary behavior and well-being outcomes are mixed [23]. Although many of the included studies were pilot or feasibility trials, they highlighted the potential for mHealth interventions to improve health behavior and health outcomes among cancer survivors. All but one study included in this review relied on self-report measures of physical activity [24]. Self-report measures have known limitations, such as recall bias; misinterpretation of items; and overestimation of activity relative to direct measures, such as accelerometer devices [25-28]. Relative to accelerometers, error rates between 35% and 79% have been observed on self-report measures [29,30]. Furthermore, it is noteworthy that only two of the included digital interventions purposively sampled cancer survivors with overweight or obesity [31,32]. This population is arguably most in need of intervention, and health behavior change interventions using nondigital mode of delivery (MOD) can improve outcomes for this cohort of survivors [33,34]. Overall, there is a need for more large-scale RCTs to provide high-quality evidence regarding the impact of mHealth interventions on objectively measured outcomes in cancer survivors with overweight or obesity.

### Aims and Objectives

The aim of this study is to investigate the impact of a personalized mHealth behavior change intervention on physical and psychological health outcomes in a group of cancer survivors with overweight or obesity. More specifically, this project examined the impact of lifestyle education and personalized goal setting, compared with standard medical care, on physical activity (step count) as well as other behavioral, clinical, and psychological outcomes.

## Methods

### Overview

The full methodological details of the trial, including a detailed description of the development of the intervention, are reported in the study protocol [35] and are summarized below. We used the eHealth extension of the CONSORT (Consolidated Standards of Reporting Trials) statement when writing this paper [36].

### Trial Design

A 2-arm, parallel, open-label RCT design was used to investigate the impact of the intervention versus standard care on clinical, psychological, and health behavior outcomes.

### Sample Size

The statistical program G*Power was used to conduct power analysis. With 2 groups (intervention and control), 3 measurements (baseline, time 1, and time 2), an assumed correlation among repeated measures of 0.3, a small-medium effect size, and a power of 0.8, the recommended sample size for repeated measures analysis of variance (ANOVA) was 102. A final sample size of 123 was calculated based on an attrition rate of 20%, as observed in similar studies using mobile technology interventions with cancer survivors [37].

### Randomization

Participants were randomized to either the intervention or the standard care control condition using a computerized random number generator (enrollment was carried out by MGK and JR, and randomization and group allocation was carried out by JG). The study was not blinded, but step count, one of the main outcome measures, was recorded directly using the Fitbit device (Healthy Metrics Research, Inc).

### Study Setting

Recruitment took place offline (by phone), and assessments were carried out face-to-face in a single hospital site, Letterkenny University Hospital, County Donegal, Ireland. Assessments were performed before randomization (T0; baseline), at 12 weeks (T1; intervention end), and at 24 weeks (T2; follow-up).

### Ethics Approval

The design of this study was approved by the Research Ethics Committee of the National University of Ireland, Galway, on September 12, 2017 (Ref: 17/MAY/20) and by the Research Ethics Committee at Letterkenny University Hospital on May 2, 2017.

### Inclusion Criteria

Adults aged 18-70 years, with a calculated BMI≥25 kg/m^2^, with a solid cancer and who had completed active cancer treatment (those continuing on endocrine therapy were permitted inclusion), who attended Oncology Services in Letterkenny University Hospital during the recruitment phase (December 2017 to January 2018), and who were willing to use mobile technology were eligible to participate.

### Recruitment

**Figure 1:**
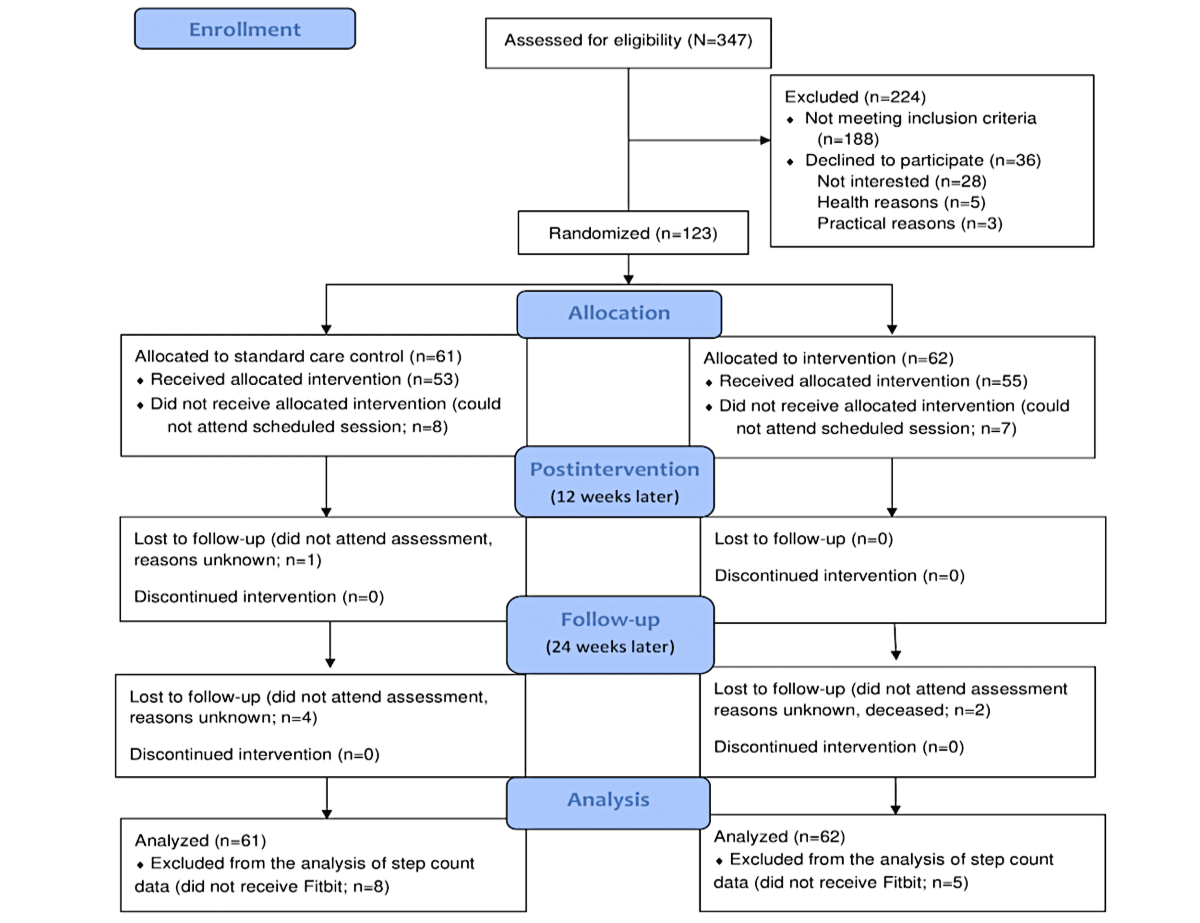
CONSORT (Consolidated Standards of Reporting Trials) diagram showing the flow of participants through each stage of the randomized controlled trial.

Participants were recruited from the Oncology Services of Letterkenny University Hospital. A total of 159 eligible participants (aged 18-70 years, BMI≥25, and active cancer treatment completed) were identified sequentially from the oncology outpatient waiting list (N=347) by the clinical team. The clinical team contacted these participants by telephone, described the aims and design of the study, and asked if they were willing to use mobile technology. Prospective participants who expressed interest in the study were sent a participant information sheet and consent form (Multimedia Appendix 1). Informed written consent was provided by 77.3% (123/159; response rate) of participants, who then underwent in-person baseline assessments. Of the 36 eligible participants who did not consent to participate (36/159, 22.6%), 28 (78%) were not interested, 3 (8%) were waiting for surgery, 1 (3%) had chronic obstructive pulmonary disease, 1 (3%) was undergoing recurrence workup, 2 (6%) had young children, and 1 (3%) did not drive (Figure 1). A total of 10 eligible participants who were willing to use mobile technology but did not own a smartphone were provided with an Amazon Fire 7 tablet.

### Intervention

This complex intervention was delivered through mHealth technology and included BCTs that aimed to improve clinical, psychological, and health behavior outcomes. The full details are described in the study protocol [35]. In summary, the intervention consisted of 2 components:

1. A 4-hour lifestyle education and information session (week 1) was delivered by health care professionals (3 physiotherapists, 1 dietician, and 1 clinical psychologist). Physiotherapists demonstrated a series of daily strengthening exercises and recommended schedules for moderate-intensity physical activity. The dietician delivered a comprehensive overview on healthy eating; answered numerous questions that clarified misinformation on nutrition; and specifically advised participants to reduce their caloric intake and reduce the intake of red meat, processed meat, salt, and sugar and increase fruit, vegetable, and fiber intake. The clinical psychologist offered practical strategies for problem solving, identifying barriers to change, and preventing relapse. The BCTs included in this session and the corresponding code from the BCT Taxonomy V1 [16] were *goal setting (outcome) (1.3), provide information on consequences of behavior to the individual (5.1), demonstration of the behavior (6.1), provide instruction on how to perform the behavior (4.1), problem solving (1.2), goal setting (behavior) (1.1),* and *action planning (1.4)*. These BCTs were applied to both physical activity and dietary behavior changes. The MOD for this component of the intervention was face-to-face human contact in real time with groups of participants. During this session, all participants were provided with a Fitbit Alta.
2. An 8-week goal-setting intervention (weeks 4-12) was delivered using mobile technology (ie, Fitbit Alta accelerometer plus SMS text messaging contact). Participants received weekly text messages with feedback on their average daily step count and a goal of increasing their step count by 10% in the following week. The BCTs included in the personalized goal-setting intervention were *self-monitoring of behavior (2.3), feedback on behavior (2.2), goal setting (behavior) (1.1), graded tasks (8.7), social reward (10.4),* and *review behavior goal(s) (1.5)*. The MOD for this intervention component was human contact at a distance using nonautomated SMS text messages facilitated using digital wearable technology (ie, Fitbit Alta). Participants continued to wear the Fitbit for the remainder of the study (24 weeks), but the personalized goal-setting intervention ceased at 12 weeks.

### Control

Participants randomized to the control condition received standard care and were also provided with a Fitbit Flex 2 to measure physical activity for the 24 weeks of the study. As such, a number of BCTs were also present in the control condition in this study. On being enrolled in the study for meeting eligibility criteria (BMI≥25 kg/m^2^), all participants were encouraged to maintain a healthy weight (*goal setting [outcome; 1.3])*. Fitbit accelerometers were distributed at a 15-minute group meeting, where provision of health information was available in the form of leaflets (information on consequences of behavior to the individual [5.1] but not BCT instruction on how to perform the behavior [4.1] or demonstration of the behavior [6.1]). In contrast to the Fitbit Alta distributed to the intervention group, the display panel on the Fitbit Flex 2 does not present summary data (ie, step count), and the app dashboard was modified to not display summary data on the participants’ mobile device. The visual display on the device and the app was limited but did not eliminate *self-monitoring of behavior* *(2.3)* in the control condition. A comparison of the BCTs included in the intervention and control conditions of the study is presented in Textbox 1.

**A comparison of the behavior change techniques included in the intervention and control conditions.**

**Intervention (Behavior Change Technique, Corresponding Code From the Taxonomy: and Definition):**

- Goal setting (outcome; 1.3): “set or agree on a goal defined in terms of a positive outcome of the wanted behavior.”
- Provide information on the consequences of behavior to the individual (5.1): “provide information (eg, written, verbal, and visual) about health consequences of performing the behavior.”
- Demonstration of the behavior (6.1): “provide an observable sample of the performance of the behavior.”
- Provide instruction on how to perform the behavior (4.1): “advise or agree on how to perform the behavior.”
- Problem solving (1.2): “analyze, or prompt the person to analyze, factors influencing the behavior and generate or select strategies that include overcoming barriers and/or increasing facilitators.”
- Goal setting (behavior; 1.1): “set or agree on a goal defined in terms of the behavior to be achieved.”
- Action planning (1.4): “prompt detailed planning of performance of the behavior, must include at least one of context, frequency, duration, and intensity.”
- Self-monitoring of behavior (2.3): “establish a method for the person to monitor and record their behavior(s) as part of a behavior change strategy.”
- Feedback on behavior (2.2): “monitor and provide informative or evaluative feedback on performance of the behavior and must include one of form, frequency, duration, and intensity.”
- Goal setting (behavior; 1.1): “set or agree on a goal defined in terms of the behavior to be achieved.”
- Graded tasks (8.7): “set easy-to-perform tasks, making them increasingly difficult, but achievable, until behavior is performed.”
- Social reward (10.4): “arrange verbal or nonverbal reward if and only if there has been effort and/or progress in performing the behavior (includes *positive reinforcement*).”
- Review behavior goal(s) (1.5): “review behavior goal(s) jointly with the person and consider modifying the goal(s) or behavior change strategy in light of achievement.”

**Control (Behavior Change Technique, Corresponding Code From the Taxonomy: and Definition):**

- Goal setting (outcome) (1.3): “set or agree on a goal defined in terms of a positive outcome of the wanted behavior.”
- Provide information on consequences of behavior to the individual (5.1): “provide information (eg, written, verbal, and visual) about health consequences of performing the behavior.”
- Self-monitoring of behavior (2.3): “establish a method for the person to monitor and record their behavior(s) as part of a behavior change strategy.”

### Materials

All participants were provided with a Fitbit activity tracker for the duration of the study. Each participant was registered with a Fitbit user account. Accounts were set up using a centralized email address corresponding to their study ID number and a randomly generated alphanumeric password. The Fitbit was set up and paired with the participants’ mobile devices (ie, smartphone or tablet). The participants were also given an information sheet with instructions on how to synchronize their Fitbit device and app and asked to perform this weekly to prevent loss of data. This sheet also contained the contact details of the research team should they encounter any technical issues or wish to discuss any concerns with their health care providers. A computer program was developed by the Insight Centre for Data Analytics at the National University of Ireland, Galway, to allow participants’ physical activity data to be extracted from the Fitbit server. A member of the research team (JG) logged in to each participant’s user account and authorized this third-party program to access their data from Fitbit. The anonymized data for all participants were exported to Excel for analysis.

### Fixes

To facilitate the goal-setting intervention, a weighted average for daily step count was calculated for participants with at least five observations per week. Participants who showed no activity for more than 2 days a week were contacted to verify that there were no technical issues. There were a number of possible reasons for someone to have 0 steps on a given day (eg, the participant did not wear the monitor or the Fitbit failed to record). These reasons were not recorded, and self-reported adherence to monitor wear was not measured. Within 2 weeks of receipt, a number of participants reported challenges using their Fitbit. As a result, all participants were invited to attend 1 of the 2 technical support sessions. A total of 12 participants attended a session and received hands-on support and troubleshooting advice regarding their device from the research team (JG and MGK). Following the implementation of the European Union General Data Protection Regulation (May 2018), participants were automatically logged out of their Fitbit app. However, this was possible to fix at a distance over the phone or via a text message.

### Outcomes

#### Clinical Outcomes

##### Anthropometric Measurements

Anthropometric measurements included weight in kilograms, BMI, and waist circumference in centimeters.

##### Functional Exercise Capacity

The 6-minute walk test measures the distance walked in 6 minutes on a hard, flat surface. Systolic blood pressure, diastolic blood pressure, heart rate, blood oxygen saturation, subjective fatigue, and dyspnea were measured pretest (ie, resting), posttest, and 4 minutes later (ie, recovery).

#### Psychological Outcomes

Health-related quality of life was measured using the Medical Outcomes Survey Short Form (RAND-36) [38]. Other measures of psychological well-being outcomes include the 3-item Loneliness Scale [39], the Brief Fatigue Inventory [40], and the General Self-Efficacy Scale [41]. Exercise self-efficacy [42] and social support for physical activity [43] were also measured.

#### Health Behavior Outcomes

Self-reported physical activity was measured using the Godin Leisure-Time Exercise Questionnaire [44], and physical activity level (ie, average daily step count) was measured directly using the Fitbit activity tracker. Dietary data were collected using the European Prospective Investigation into Cancer and Nutrition Norfolk Food Frequency Questionnaire [45].

All outcomes were measured at baseline (T0), 12 weeks (T1; intervention end), and 24 weeks (T2; follow-up). The measures are described in full in the trial protocol [35].

### Statistical Methods

#### Missing Data

To maximize power and conform to intent-to-treat analysis, missing data were handled using the expectation-maximization (EM) algorithm. A nonsignificant MCAR test [46] showed that the data were missing completely at random (*χ^2^_30,080_*=113.3 *P*=.99); therefore, data substitution methods were deemed appropriate. For step count data specifically, EM data substitution was applied only to the 107 participants who received a Fitbit. The 16 participants in the intent-to-treat group (ie, those who could not attend the initial session where Fitbits were distributed) were not included in the missing value analysis and EM data substitution for the analyses of group differences in step count.

#### Analysis

A series of 3 (time: baseline [T0], 12 weeks [T1], and 24 weeks [T2])×2 (group: control and intervention) mixed ANOVAs were performed to determine the effect of the intervention on clinical, psychological, and health behavior outcomes. In the case of a significant interaction effect, follow-up two-tailed independent sample *t* tests were conducted to investigate between-group differences at each time point, and one-way ANOVAs were conducted to identify within-group differences across time points. Independent samples *t* tests were used to analyze group differences (control and intervention) in average daily step count across the 24 weeks of the study.

## Results

### Participant Flow

A flow diagram of the progress through each phase of this 2-group parallel randomized trial is shown in Figure 1. A total of 123 eligible participants underwent baseline assessments. The participants were then randomized to the control or intervention arm. Of the 123 participants, 62 (50.4%) were assigned to the intervention group and were invited to attend a lifestyle education and information session where they would also receive their Fitbit activity monitor, and out of these, 55 (89%) participants were able to attend the session. The remaining 61 participants assigned to the control group were invited to an appointment where they were provided with a Fitbit activity monitor, and out of these, 53 (87%) participants were able to attend the session. All participants were invited to a postintervention assessment (12 weeks later) to determine the impact of the lifestyle education and information session and personalized goal-setting mHealth intervention on improving clinical, psychological, and health behavior outcomes. A total of 55 participants in the intervention condition and 52 participants in the control condition attended the assessment. Finally, to determine whether any effects of the intervention were maintained 3 months later, all participants were invited to a follow-up assessment (24 weeks after baseline assessment). In total, 53 participants in the intervention group and 48 participants in the control group attended the follow-up assessment. This resulted in an overall attrition rate of 21% (13/61) in the control arm and 14% (9/62) in the intervention arm.

### Baseline Data

Participants’ characteristics are described in Table 1. Randomization resulted in an intervention group that was younger, had lower weight and BMI, and had a higher number of males.

**Table 1:** Participants’ characteristics at baseline assessment (N=123).

Characteristics		Control (n=61)	Intervention (n=62)
Age (years), mean (SD)		59.24 (7.65)	55.61 (8.05)
Weight (kg), mean (SD)		87.10 (16.32)	84.18 (13.98)
BMI (kg/m^2^), mean (SD)		32.64 (5.41)	30.33 (3.99)
**Gender, n**			
	Female	49	42
	Male	4	12
**Cancer diagnosis, n (%)**			
	Breast	49 (80)	50 (81)
	Prostate	1 (2)	1 (2)
	Lung	0 (0)	1 (2)
	Colorectal	9 (15)	8 (13)
	Testicular	2 (3)	2 (3)
**Have you ever been told by a doctor that you have or have had any of the following conditions? n (%)**			
	Angina	1 (2)	2 (4)
	Heart attack	3 (6)	1 (2)
	High blood pressure	19 (36)	18 (33)
	Stroke	3 (6)	1 (2)
	Diabetes	5 (9)	6 (11)
	High cholesterol	21 (40)	20 (37)
	Depression	12 (23)	9 (17)
	Anxiety	12 (23)	12 (22)

### Clinical Outcomes

#### Anthropometric Measurements

Means and SDs for all anthropometric measurements are presented in Table 2.

**Table 2:** Anthropometric measurements.

Outcome	Weight, mean (SD)			BMI, mean (SD)				Waist circumference, mean (SD)		
	T0^a^	T1^b^	T2^c^	T0	T1	T2	T0		T1	T2
Control	86.9 (15.2)	85.99 (14.94)	86.16 (14.76)	32.44 (5.07)	32.26 (5.02)	32.33 (5.03)	103.35 (9.28)		101.82 (9.41)	101.53 (9.38)
Intervention	84.17 (13.11)	82.11 (13.41)	82.59 (13.69)	30.47 (3.74)	29.78 (4.04)	29.95 (4.12)	101.15 (11.07)		98.43 (11.72)	98.13 (11.69)

^a^T0: time 0 (baseline).

^b^T1: time 1 (intervention end; 12 weeks).

^c^T2: time 2 (follow-up; 24 weeks).

##### Weight

There was no significant interaction effect on weight (*F*_2,242_=2.615; *P*=.07). A main effect of time was observed (*F*_2,242_=18.14; *P*<.001; *ηp^2^*=0.13). There was no main effect of group (F_1,121_=1.786; *P*=.18).

##### BMI

**Figure 2:**
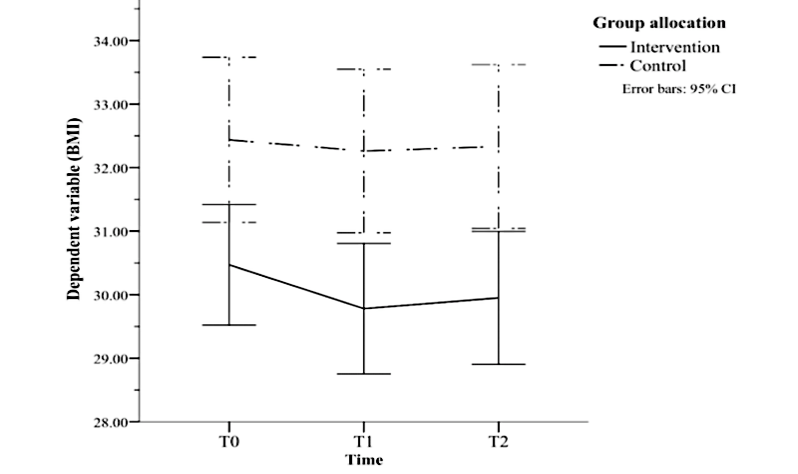
Results of 3×2 mixed analysis of variance showing a significant reduction in BMI for the intervention group only. T0: time 1, baseline; T1: time 1, intervention end (12 weeks); T2: time 2, follow-up (24 weeks).

There was a significant interaction between group and time (*F*_2,242_=4.149; *P*=.02; *ηp^2^*=0.033) as shown in Figure 2. Follow-up *t* tests revealed significant group differences in BMI at baseline (*t_121_*=2.451; *P*=.02), at 12 weeks (*t_121_*=3.018; *P*=.003), and at 24 weeks (*t_121_*=2.876; *P*=.005). There was a significant change in BMI across time points in the intervention group (*F*_2,122_=12.513; *P*<.001; *ηp^2^*=0.17). BMI was significantly lower at both 12 weeks (mean difference [MD] −0.689; *P*<.001) and 24 weeks (MD −0.520; *P*=.007) than at baseline. In the control group, there was a nonsignificant reduction in BMI (*F*_2,120_=1.041; *P*=.36) at 12 weeks (MD −0.18) and 24 weeks (MD 0.11).

##### Waist Circumference

**Figure 3:**
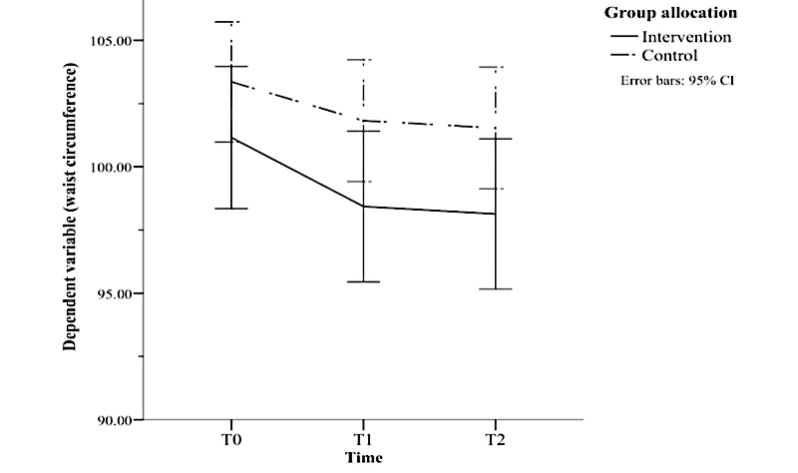
Results of 3×2 mixed analysis of variance showing a significant reduction in waist circumference that was maintained at follow-up in both conditions, with a larger reduction in the intervention group. T0: time 1, baseline; T1: time 1, intervention end (12 weeks); T2: time 2, follow-up (24 weeks).

There was a significant interaction effect for waist circumference (*F*_2,242_=3.342; *P*=.04; *ηp^2^*=0.027) shown in Figure 3. Post hoc analysis revealed a significant change in waist circumference across time points in both the intervention group (*F*_2,122_=29.632; *P*<.001; *ηp^2^*=0.327) and the control group (*F*_2,120_=20.08; *P*<.001; *ηp^2^*=0.251). In the intervention group, waist circumference was significantly lower at both 12 weeks (MD −2.725; *P*<.001) and 24 weeks (MD −3.019; *P*<.001) than at baseline. This trend was also observed in the control group, with waist circumference significantly lower at both 12 weeks (MD −1.535; *P*<.001) and at 24 weeks (MD −1.822; *P*<.001) than at baseline. However, the magnitude of change was greater in the intervention group than in the control group (MD −1.19, SD 0.56; 95% CI −2.31 to −0.06; *t_121_*=2.091; *P*=.04). The difference in waist circumference between 12 and 24 weeks was not significant in the intervention group (MD −0.294; *P*=.17) or the control group (MD −0.286; *P*=.21).

##### Functional Exercise Capacity

There was no significant interaction effect for distance walked; systolic blood pressure; diastolic blood pressure; heart rate; subjective fatigue; or dyspnea measured before, after, or 3 minutes after the 6-minute walk test (the full set of results are presented in Multimedia Appendix 2). In short, the main effects of time showed that both the groups significantly improved from baseline to T2 in 13 measures of functional exercise capacity (all *P*<.001), and the means and SDs are presented in Table 3.

**Table 3:** Measures of the 6-minute walk test.

Outcomes	Control, mean (SD)			Intervention, mean (SD)		
	T0^a^	T1^b^	T2^c^	T0	T1	T2
Distance walked	515.99 (67.9)	551.25 (62.44)	566.29 (71.79)	532.57 (69.8)	571.72 (61.68)	590.28 (87.78)
Resting SBP^d^	144.12 (19.57)	135.3 (16.16)	139.45 (13.24)	139.95 (21.04)	129.97 (16.27)	139.99 (33.52)
Posttest SBP	154.48 (20.83)	151.03 (19.18)	150.66 (17.33)	151.67 (23.43)	145.1 (21.07)	152.97 (38.02)
Recovery SBP	142.32 (17.66)	134.31 (17.25)	135.08 (14.74)	138.01 (18.2)	129.38 (15.96)	132.81 (19.19)
Resting DBP^e^	80.89 (7.86)	76.76 (9.2)	80.67 (8.25)	79.56 (9.79)	76.11 (9.12)	77.54 (11.06)
Posttest DBP	82.15 (8.94)	81.78 (12.17)	81.89 (11.66)	81.27 (12.45)	77.39 (10.75)	80.28 (10.83)
Recovery DBP	80.27 (8.06)	77.54 (9)	78.31 (7.45)	78.83 (10.62)	76.35 (9.32)	78.28 (9.74)
Resting HR^f^	79.01 (10.28)	77.36 (8.56)	77.4 (9.27)	78.73 (11.4)	77.56 (15.17)	74.09 (13.30)
Posttest HR	111.67 (17.39)	103.51 (17.94)	113.45 (17.61)	127.06 (143.76)	105.89 (24.44)	113.53 (50.21)
Recovery HR	85.49 (11.88)	82.07 (10.77)	83.5 (10.75)	85.13 (13.32)	82.31 (12.45)	83.02 (15.57)
Resting fatigue	6.39 (0.9)	6.22 (0.91)	5.96 (1.06)	6.35 (0.79)	6.17 (1.03)	6.05 (1.39)
Posttest fatigue	9.83 (2.36)	10.73 (1.98)	10.35 (2.72)	9.9 (2.41)	10.54 (2.45)	10.2 (3.19)
Recovery fatigue	6.93 (1.6)	6.4 (0.98)	6.09 (1.42)	6.88 (1.23)	6.56 (1.38)	6.08 (2.71)
Resting dyspnea	1.24 (0.73)	1.12 (0.42)	1.58 (1.19)	1.19 (0.65)	1.15 (0.67)	1.35 (0.97)
Posttest dyspnea	4.02 (1.67)	3.74 (1.47)	5.06 (4.61)	3.5 (1.33)	4.04 (1.75)	4.25 (2.4)
Recovery dyspnea	1.81 (0.97)	1.24 (0.47)	1.88 (1.88)	1.64 (0.94)	1.40 (0.87)	1.45 (1.51)

^a^T0: time 0 (baseline).

^b^T1: time 1 (intervention end; 12 weeks).

^c^T2: time 2 (follow-up; 24 weeks).

^d^SBP: systolic blood pressure.

^e^DBP: diastolic blood pressure.

^f^HR: heart rate.

#### Psychological Outcomes

##### Quality of Life

No significant interaction effects were observed for health-related quality of life (measured by the RAND36 Medical Outcomes Survey). Means and SDs are presented in Table 4, and the results of the ANOVA are presented in Multimedia Appendix 3.

**Table 4:** Subscales of RAND-36 Medical Outcomes Survey.

Outcome	Control, mean (SD)				Intervention, mean (SD)		
	T0^a^	T1^b^	T2^c^	T0		T1	T2
Physical functioning	77.87 (17.06)	81.81 (14.52)	81.15 (17.28)	73.87 (20.33)		80.08 (17.28)	77.66 (19.83)
Role limitations: physical health	69.67 (39.82)	78.28 (29.39)	77.05 (35.15)	69.76 (37.64)		85.89 (30.57)	79.03 (33.63)
Role limitations: emotional health	83.06 (33.12)	89.62 (23.99)	83.06 (32.56)	72.58 (40.72)		87.37 (29.37)	83.33 (31.22)
Pain	73.24 (21.85)	78.11 (21.65)	74.22 (23.63)	73.47 (23.16)		79.28 (19.62)	75.57 (21.52)
Emotional well-being	77.38 (17.57)	81.77 (13.59)	83.74 (13.28)	73.03 (18.66)		79.87 (16.32)	78.77 (18.04)
Social functioning	78.48 (23.4)	91.8 (15.79)	88.31 (18.8)	78.63 (23.01)		89.31 (17.8)	85.88 (20.69)
Energy	61.39 (20.21)	70.99 (16.38)	68.69 (19.68)	53.39 (21.27)		67.02 (17.15)	63.06 (18.07)
General health	51.73 (20.17)	57.6 (15.87)	55.87 (16.4)	49.5 (16.54)		56.98 (12.94)	53.04 (15.74)

^a^T0: time 0 (baseline).

^b^T1: time 1 (intervention end; 12 weeks).

^c^T2: time 2 (follow-up; 24 weeks).

##### Other Psychological Outcomes

There were also no significant interaction effects for loneliness, self-efficacy, exercise self-efficacy, or exercise social support (full results, including nonsignificant findings, are presented in Multimedia Appendix 4). The means and SDs are presented in Table 5.

**Table 5:** Psychological outcome measures.

Outcomes	Control, mean (SD)				Intervention, mean (SD)		
	T0^a^	T1^b^	T2^c^	T0		T1	T2
Loneliness	4.23 (1.64)	4.1 (1.57)	4.34 (1.63)	3.63 (1.02)		3.69 (0.9)	3.74 (1.01)
Fatigue (global)	35.47 (20.47)	25.18 (20.79)	31.05 (20.3)	23.36 (19.42)		20 (16.45)	21.49 (15.95)
Fatigue severity	9.77 (4.92)	8.05 (4.88)	8.9 (4.59)	6.92 (3.89)		6.38 (3.55)	6.90 (3.47)
Fatigue interference	20.63 (14.43)	13.28 (14.64)	17.76 (14.21)	13.1 (13.47)		10.53 (11.33)	11.48 (10.99)
Self-efficacy	20.56 (4.45)	22.27 (4.26)	21.96 (4.62)	21.69 (4.19)		22.16 (3.92)	22.01 (4.02)
Exercise: self-efficacy	22.33 (4.14)	21.93 (4.56)	21.08 (4.98)	23.14 (2.49)		22.69 (3.85)	22.41 (3.63)
Exercise: social support	12.36 (5.39)	13.39 (4.39)	12.45 (4.8)	12.33 (4.92)		13.03 (4.31)	12.1 (5.01)

^a^T0: time 0 (baseline).

^b^T1: time 1 (intervention end; 12 weeks).

^c^T2: time 2 (follow-up; 24 weeks).

##### Fatigue

**Figure 4:**
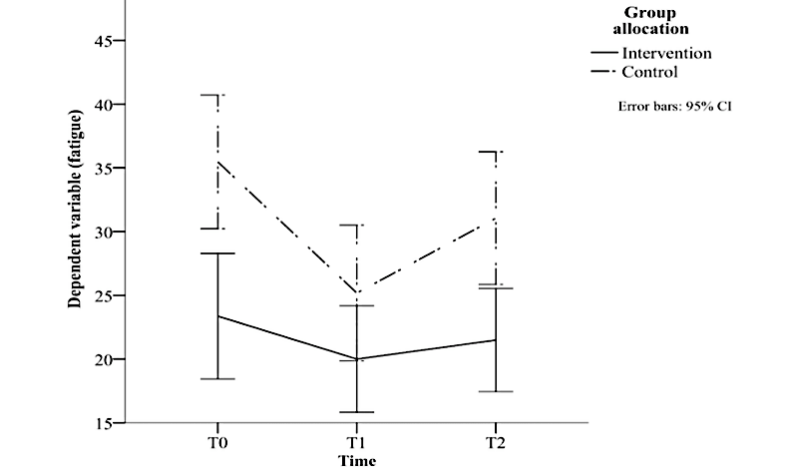
Results of a 3×2 mixed analysis of variance showing a significant reduction in fatigue at 12 weeks and a nonsignificant increase at 24 weeks in the control group only. T0: time 1, baseline; T1: time 1, intervention end (12 weeks); T2: time 2, follow-up (24 weeks).

As shown in Figure 4, there was a significant interaction between groups and time (*F*_2,242_=3.199; *P*=.04; *ηp^2^*=0.026). Independent samples *t* tests revealed significant differences between the intervention and control groups at baseline (*t_121_*=3.365; *P*=.001) and at 24 weeks (*t_121_*=2.908; *P*=.004) but not at 12 weeks (*t_121_*=1.534; *P*=.13). Fatigue remained stable in the intervention group (*F*_2,122_=1.815; *P*=.17). The change in global fatigue in the control group was significant (*F*_2,120_=11.701; *P*<.001; *ηp^2^*=0.163). Fatigue was significantly lower at 12 weeks than at baseline (MD −10.289; *P*<.001) and was significantly higher at 24 weeks than at 12 weeks (MD 5.872; *P*=.001). The difference in fatigue between baseline and 24 weeks was not significant (MD −4.417; *P*=.052).

#### Health Behavior Outcomes

##### Dietary Behavior

Dietary data were collected using the European Prospective Investigation into Cancer and Nutrition Norfolk Food Frequency Questionnaire 45. There were no significant interaction effects for any of the 10 food groups assessed (Multimedia Appendix 5), and the means and SDs are presented in Table 6.

**Table 6:** Dietary behavior.

Outcomes	Control, mean (SD)				Intervention, mean (SD)		
	T0^a^	T1^b^	T2^c^	T0		T1	T2
Fiber	16.25 (4.06)	16.33 (4.78)	16.64 (4.14)	17.63 (6.62)		17.45 (5.32)	18.6 (6.18)
Kilocalorie	1846.14 (538.65)	1681.15 (562.66)	1687.54 (455.96)	2030.77 (664.09)		1760.36 (569.79)	1828.44 (687.56)
Sodium	2940.68 (891.54)	2547.2 (785.47)	2685.03 (759.71)	3131.63 (1021.24)		2586.32 (834.46)	2840.5 (1075.67)
Saturated fats	29.06 (13.26)	24.91 (10.35)	26.30 (10.29)	31.95 (14.17)		24.80 (13.1)	27.52 (15.98)
Fruit	243.29 (138.28)	291.02 (153.8)	269.74 (160.84)	259.69 (179.98)		310.94 (214.98)	312.33 (329.19)
Meat	126.40 (54.37)	103.33 (48.28)	119.18 (49.56)	125.58 (62.87)		106.49 (78.39)	120.22 (59.21)
Sugar	48.65 (45.42)	31.84 (28.06)	35.55 (28.4)	55.51 (57.16)		31.09 (22.25)	36.89 (36.42)
Vegetables	231.91 (101.65)	244.62 (95.77)	263.57 (105.37)	248.9 (127.94)		264.88 (106.22)	303 (130.75)
Alcohol	2.02 (2.67)	1.75 (2.46)	2.15 (2.6)	3.62 (9.97)		2.80 (7.23)	3.28 (7.95)
Alcoholic beverages	22.53 (27.63)	20.67 (28.95)	27.79 (39.86)	44.97 (132.53)		31.81 (91.21)	40.67 (108.43)

^a^T0: time 0 (baseline).

^b^T1: time 1 (intervention end; 12 weeks).

^c^T2: time 2 (follow-up; 24 weeks).

##### Self-reported Physical Activity Level

There was no significant main effect of time (*F*_2,242_=1.56; *P*=.21). There was no main effect of condition (*F*_1,121_=0.073; *P*=.79) and no significant interaction effect (*F*_2,242_=0.260; *P*=.77). The means and SDs are presented in Table 7.

**Table 7:** Scores on the Godin Leisure-Time Exercise Questionnaire.

Outcome	Control, mean (SD)				Intervention, mean (SD)		
	T0^a^	T1^b^	T2^c^	T0		T1	T2
Weekly leisure activity	31.03 (17.25)	34.68 (26.12)	33.03 (29.39)	31.14 (20.52)		34.75 (18.34)	30.38 (16.71)

^a^T0: time 0 (baseline).

^b^T1: time 1 (intervention end; 12 weeks).

^c^T2: time 2 (follow-up; 24 weeks).

##### Direct Physical Activity Level

The step count data were collected continuously using Fitbit. Daily step count totals were summed, and an average daily step count was calculated for each week of the 24-week study. Means and SDs are presented in Table 8, along with the results of independent *t* tests comparing group differences in step count. The intervention group had a significantly higher average daily step count on 13 of the 24 weeks of the study (ie, weeks 3, 5-9, 11, 12, 14-17, and 21), contributing to an additional 1689-2500 steps per day (mean 2032, SD 270).

An analysis of the personalized goal-setting intervention demonstrated that 69% (37/54) of participants in the intervention group met at least 50% (4/8) of their step count goals. However, the goal success rate was not significantly correlated with any of the study outcome variables. A further analysis of prescribed step count goals within the context of goal achievement indicates that success was highest in the earlier stages of the goal-setting intervention when step count goals were below 10,000 steps (Table 9).

**Table 8:** Average daily step count for weeks 1-24 (N=107).

Group	Control, mean (SD)	Intervention, mean (SD)	*t* test (*df*)	*P* value
Week 1	8792.11 (3990.17)	8775.34 (8946.51)	0.012 (105)	.99
Week 2	8434.01 (4135.28)	8978.65 (6063.41)	−0.542 (105)	.59
Week 3	8622.72 (3126.16)	10,401.04 (3659.81)	−2.7 (105)	.01
Week 4 (SMS 1)	8638.9 (3768.52)	9833.51 (3737.18)	−1.646 (105)	.10
Week 5 (SMS 2)	8359.62 (3414.64)	10,621.07 (4020.28)	−3.133 (105)	<.001
Week 6 (SMS 3)	8308.83 (3523.38)	10,265.19 (4345.32)	−2.555 (105)	.01
Week 7 (SMS 4)	8519.34 (3318.01)	10,982.03 (4708.59)	−3.122 (105)	<.001
Week 8 (SMS 5)	9940.19 (4317.15)	12,247.11 (5842.16)	−2.319 (105)	.02
Week 9 (SMS 6)	8819.53 (4088.03)	10,744.1 (4681.16)	−2.263 (105)	.03
Week 10 (SMS 7)	9065.88 (3755.52)	10,262.84 (4780.74)	−1.438 (105)	.15
Week 11 (SMS 8)	8576.29 (3865.43)	10,610.33 (5441.93)	−2.225 (105)	.03
Week 12	9067.76 (3838.9)	10,915.87 (4804.31)	−2.196 (105)	.03
Week 13	9883.54 (3462.85)	11,080.53 (5621.11)	−2.113 (105)	.19
Week 14	9219.04 (3711.7)	10,908.43 (4703.33)	−2.06 (105)	.04
Week 15	9185.22 (3715.93)	11,260.69 (4871.56)	−2.474 (105)	.01
Week 16	7750.5 (3536.26)	9553.78 (5119.87)	−2.116 (105)	.04
Week 17	8726.35 (3938.05)	10,513.67 (4666.4)	−2.139 (105)	.04
Week 18	9020.32 (3487.54)	10,255.48 (3446.08)	−1.843 (105)	.07
Week 19	9480.41 (3820.46)	10,901.42 (4965.92)	−1.657 (105)	.10
Week 20	8624.73 (3426.48)	10,108.47 (4817.34)	−1.833 (105)	.07
Week 21	7700.75 (4584.27)	10,201.34 (4386.2)	−2.883 (105)	<.001
Week 22	7520.26 (11826.73)	10,171.88 (7265.27)	−1.4 (105)	.16
Week 23	8224.2 (5145.45)	9481.54 (5442.37)	−1.228 (105)	.22
Week 24	8483.63 (4808.37)	9700.24 (5860.72)	−1.173 (105)	.24

**Table 9:** Average daily step count goal for each week and participants’ rate of success in achieving step count goals in weeks 5-12 (n=54).

Time	Goal 1: week 5	Goal 2: week 6	Goal 3: week 7	Goal 4: week 8	Goal 5: week 9	Goal 6: week 10	Goal 7: week 11	Goal 8: week 12
Step count goal, mean (SD)	7541.75 (4046.05)	7907.88 (5186.28)	10,601.11 (4874.16)	11,598.83 (5337.32)	9396.26 (5745.74)	10,922.66 (6074.49)	11,145.08 (5479.19)	11,294.56 (6039.08)
Did achieve goal, n (%)	41 (76)	39 (72)	25 (46)	35 (65)	35 (65)	18 (33)	17 (32)	24 (44)
Did not achieve goal, n (%)	13 (24)	15 (28)	29 (54)	19 (35)	19 (35)	36 (67)	37 (68)	30 (56)

## Discussion

### Principal Findings

The aim of this trial is to examine the impact of a personalized mHealth behavior change intervention on clinical, psychological, and health behavior outcomes among a group of cancer survivors with overweight or obesity. The results show that the intervention yielded several significant benefits over and above that shown in the standard care control group. The intervention group had a significantly greater reduction in BMI than the control group. This reduction in BMI was maintained at the 24-week follow-up. Relative to the control group, there was a significantly greater reduction in waist circumference in the intervention group. At follow-up, there was a modest reduction in BMI (0.52) and waist circumference (3.02 cm) with small to medium effect sizes. In relation to behavioral outcomes, participants in the intervention group had significantly higher physical activity during both the intervention phase (8 out of the 12 weeks) and the follow-up phase (5 out of the 12 weeks) than those in the control group. Participants in the intervention averaged approximately 2000 extra steps per day (the equivalent of 1 mile or 20 minutes of physical activity [47]). However, there were no significant changes in functional exercise capacity, dietary behavior, or psychological outcomes.

The design of this intervention is aligned with the National Institute for Health and Care Excellence guidelines for weight management in people with obesity [48]. They recommend multicomponent interventions that include effective behavior change strategies aimed at increasing physical activity or decreasing sedentary behavior, reducing energy intake, and improving diet quality. Although the statistically significant changes in anthropometric measures observed in this study fall short of the 5% change deemed clinically significant, the clinical guidelines for obesity management acknowledge that more modest losses can also yield significant health benefits [48]. The magnitude of change observed here is in line with the results of traditional behavioral interventions for people with overweight or obesity [49] and cancer [50]. They are also consistent with interventions using digital MOD for people with chronic conditions, such as obesity [51] and cancer [23]. Cancer survivorship is characterized by ongoing physical and psychological challenges, making behavior change and weight management more difficult [52], and any change observed is of paramount clinical importance for this population. Furthermore, the results of a mixed methods investigation nested within this RCT and published separately concluded that this intervention is acceptable to participants [53], in addition to being effective.

A key element of this intervention was the use of personalized goals delivered through mobile technology (ie, Fitbit and SMS text messaging contact). This aimed to enhance participants’ motivation to increase their overall levels of physical activity. In addition to significant increases in physical activity (step count), the results show a good level of goal achievement, suggesting that goal-setting intervention did influence participants’ motivation to increase activity. Although goals were personalized (ie, increase daily step count by 10% per week), goal attainment was highest in the initial stages when step count targets were lower, suggesting there may be a threshold after which increasing step count become unattainable. This is not surprising, and recent evidence suggests that significant health benefits are achievable at much lower levels of physical activity (ie, 4400-7500 steps per day) [54]. This is also in line with existing studies, which found that setting a higher physical activity goal leads to higher physical activity levels but concurrently lower goal achievement [55]. This suggests that goal setting may be more effective in the early stages of the physical activity intervention, but it becomes less critical to attaining a higher physical activity level once a certain threshold is consistently achieved or perhaps when health behaviors have been consolidated. If one assumes that a low level of goal achievement is deleterious over time (eg, decrease in motivation) [56], the results do not rule out the existence of an *optimal goal-setting zone* for some participants. This was the approach taken in this study, where targets were based on current performance. Swann et al [56] argued that achievement of goals is actually not the primary aim of goal-setting theory; instead, goal setting is simply a mechanism for enhancing task performance regardless of whether the goal is achieved. Within this context, a higher step count is a positive outcome, despite failure to achieve step count targets at the higher end of the spectrum. It is likely that this component of the intervention, including the highly effective BCTs of *goal setting, feedback, review,* and *self-monitoring* [17-19] delivered via mobile technology, facilitated the higher levels of physical activity in the intervention participants, thus contributing to the significant reductions in BMI and waist circumference.

Although the results did not demonstrate any significant improvements in dietary behavior, it is clear that changes in lifestyle (primarily increased physical activity) contributed to significantly greater benefits in key clinical outcomes for the intervention group. An emerging body of research with cancer survivors suggests that digital interventions have positive effects on BMI and physical activity, but the findings are less consistent for diet [23]. For example, the digital health behavior change intervention by O’Carroll Bantum et al [57] also found increased physical activity but no increase in fruit or vegetable intake among cancer survivors. As such, the results of this study are in line with those of previous studies. On reflection it is not wholly unexpected that no significant dietary behavior changes were found. Participants in the intervention group attended a 4-hour lifestyle education and information session that included BCTs related to dietary behavior change delivered by a dietician, in addition to a goal-setting intervention that focused exclusively on physical activity. This may not have been sufficient to change dietary behavior. In postintervention interviews, participants indicated that additional behavioral support was needed to change their diet [53]. The success of the goal-setting intervention for increasing step count in this study is encouraging, and future digital interventions should consider goal setting in relation to dietary behavior in combination with physical activity goals.

This intervention had 2 behavioral targets (increase physical activity and improve diet) to improve health and well-being outcomes. Systematic review evidence has found that health behavior change interventions focusing on physical outcomes improve well-being in the general population [8] and among cancer survivors [9,10]. Therefore, it was unexpected that despite increased physical activity levels, the intervention group did not report significant improvements in any of the psychological well-being outcomes over the course of the study. These findings are consistent with other lifestyle interventions for cancer survivors that also found no significant group differences in quality of life [32,58,59], well-being [23], or fatigue [20,60]. One possibility for the lack of significant effects is the relatively high levels of well-being in participants at baseline. Furthermore, although participants received a comprehensive presentation from a clinical psychologist at the lifestyle education and information session, this presentation focused on behavior (eg, action planning and problem solving); thus, there was no aspect of the intervention that deliberately targeted increased well-being. Future interventions may wish to incorporate techniques and strategies aimed at improving well-being more directly. Nevertheless, qualitative data show that participants perceived the aim of the intervention to be *moving on* psychologically from cancer and reported emotional and psychological improvements as a result of participating [53]. These self-reported improvements did not translate into statistically significant interaction effects in this trial.

### Strengths and Limitations

Although the effect sizes were small to medium, the large sample size and high retention rate means that the study was adequately powered to detect such effects. Participants were randomized to conditions to reduce selection bias, and the use of intention-to-treat analysis limited the impact of attrition bias. However, it was not possible to blind participants or outcome assessors in this study, a limitation common to many digital health interventions [36]. This may have introduced a performance or detection bias. Furthermore, the analyses were not adjusted for baseline differences that occurred due to chance. Unadjusted analyses from randomized trials provided valid estimates of effects [61], and baseline levels of variables that are likely to be prognostic (ie, anthropometric measures) were accounted for in the analyses. Nevertheless, these results should be interpreted with caution. The participants were mostly female breast cancer survivors. This is representative of trends in the wider literature [62] but may limit the generalizability of the findings to people with other types of cancer and to people of other genders in the wider population. Furthermore, inclusion criteria required participants to be willing to use mobile technology. This may have contributed to a digital divide, in the sense that prospective participants who may have benefited from the intervention were excluded because they were inexperienced or uncomfortable using digital technologies. It is worth noting that no one was excluded due to a lack of mobile technology; participants who did not have access to but were willing to engage with mobile technology were provided with a mobile device (Amazon Fire tablet) by the research team. Finally, the participants in the trial had access to technical support from the research team, as needed. This was front loaded, as support was needed more frequently at commencement when participants were becoming familiar with the technology. This may not be feasible in standard oncology care, limiting the applicability of the findings outside of an RCT setting. A custom-built front-end software was used to bulk export participants’ step count data to facilitate the weekly goal-setting intervention; as such, something similar may be needed if applying the same intervention in a health care setting [63]. Future research will be needed to identify potential implementation issues for delivering this intervention in clinical settings.

A notable strength of this study is the use of a direct measure of physical activity (ie, Fitbit accelerometer). In a review of 15 digital health behavior change interventions with cancer survivors [23], no study had used a direct measure of physical activity to evaluate the impact of the intervention. The majority of studies rely on self-report measures that are biased in a number of ways and provide less accurate estimates than accelerometers [25-28,30]. Consumer-based wearable accelerometers offer similar accuracy to criterion measures in controlled settings [64,65] and in free-living settings [66,67]. However, relative to research-grade accelerometers, some studies have reported underestimation [68] and overestimation of steps [69], especially at faster ambulatory speeds. In addition to the high cost of these devices (eg, ActivPAL and ActiGraph), there are a number of practical constraints to their use in studies with large samples and over longer time frames [70,71]. To our knowledge, no study has examined the potential differences in precision between the 2 Fitbit models used in this study. Reassuringly, the Fitbit has been consistently rated among the most accurate consumer-based wearable activity monitor for measuring step count [64,68,72]. According to the authors’ knowledge, this trial is the first to evaluate a digital health behavior change intervention using a direct measure of physical activity with a sample of cancer survivors with overweight or obesity. That being said, self-report measures of dietary behavior were used in this study, although interviews by health care professionals (ie, 24-hour dietary recall) would have been superior, particularly for accurately measuring caloric intake.

### Conclusions

Cancer survivors who have overweight or obesity require additional support to self-manage their health behaviors. mHealth technology may provide a cost-effective solution within modern oncology care. mHealth has enormous potential for improved health care delivery, but evidence from this group currently lacks a strong base [58,62]. The results of this study represent a promising contribution to the field. This mHealth intervention significantly reduced BMI and waist circumference and increased physical activity levels, but it was consistent with an emerging body of research with cancer survivors [23], which demonstrates limited impact on diet or well-being. Future research is needed to continue evaluating and refining mHealth behavior change interventions to improve health and well-being outcomes for the growing number of cancer survivors.

## Appendix

Multimedia Appendix 1 Participant information sheet and consent form.

Multimedia Appendix 2 Results of 3×2 analysis of variance on measures of the 6-minute walk test.

Multimedia Appendix 3 Results of 3×2 analysis of variance on subscales of the RAND-36 Medical Outcomes Survey: Short Form.

Multimedia Appendix 4 Results of 3×2 analysis of variance on other psychological outcomes.

Multimedia Appendix 5 Results of 3×2 analysis of variance on dietary behavior.

Multimedia Appendix 6 CONSORT-eHEALTH checklist (V 1.6.1).

## Abbreviations

ANOVA: analysis of variance
BCT: behavior change technique
CONSORT: Consolidated Standards of Reporting Trials
EM: expectation-maximization
MD: mean difference
mHealth: mobile health
MOD: mode of delivery
RCT: randomized controlled trial
