# Supplementary material for: Examining the Impact of an mHealth Behavior Change Intervention With a Brief In-Person Component for Cancer Survivors With Overweight or Obesity: Randomized Controlled Trial
Source: JMIR Mhealth Uhealth. 2021 Jul 5;9(7):e24915. doi: 10.2196/24915 (PMC8406099; doi:10.2196/24915)
Supplement: Multimedia Appendix 1 [file mhealth_v9i7e24915_app1.doc]

**Multimedia Appendix 1.** Participant information sheet and consent form.

**A trial to investigate the impact of a personalised self-management lifestyle programme using mobile technology on the health and wellbeing of cancer survivors.**

Introduction:

Thank you for taking the time to read this information sheet. You are invited to take part in a research study because you have had cancer and are attending the oncology services for follow-up. Before you decide if you would like to take part or not it is important that you understand why the research is being done and what it will involve. This *Participant Information Leaflet* tells you about the purpose, risks and benefits of this research study. If you agree to take part, we will ask you to sign a *Consent Form* which is the last page of this document. If there is anything that you are not clear about, we will be happy to explain it to you. Please take as much time as you need to read this information. You should only consent to participate in this research study when you feel you understand what is being asked of you, and you have had enough time to think about your decision. Thank you for reading this.

**What is the purpose of this study?** In Ireland an average of 37,000 new cases of cancer are diagnosed each year and the incidence is increasing. However the good news is that more people are living longer after cancer and even being cured in more than half of all cases. Cancer treatment is difficult and can affect how someone is able to get back to work, care for their loved ones or just be involved in life again. People are followed up in hospital outpatient clinics but this doesn’t always help them get their general health back on track.

We want to see if we can improve how people feel both physically and psychologically after they have had cancer. We know that being overweight increases your risk of developing cancer and it makes people feel less well overall. This study involves approaching people who have had cancer and have overweight and asking them to take part in this programme. If you do decide to participate we will invite you into the hospital to discuss the study in detail with one of the research team.

Where is this study being carried out: It is being carried out Letterkenny University Hospital, Co. Donegal. It is funded locally by the Relay for Life/Irish Cancer Society. Researchers from Letterkenny University Hospital Oncology Department and NUI Galway School of Psychology are conducting this research.

**What will happen during the study?** Once you decide to participate you will sign the consent form for this study. After that we will carry out a baseline assessment to assess your general health and fitness. You will then be randomised to either the control group or the active programme. Randomisation means that it will be decided by computer which group you will be in, much like the flip of a coin. The research team will have no say in this decision.

A Fitbit will be worn by all participants to monitor progress. It is similar to a watch and is worn on your wrist. We will follow you up in person and by text message contact for 6 months after you start the programme to see if your weight, diet, fitness and wellbeing have changed. You will be required to fill out some questionnaires about your physical activity and diet throughout the programme. You will also attend the hospital to meet with the research team at the beginning of the study, 3 months later, and at 6 months later (3 visits). Some participants will be asked to participate in a focus group or interviews at the end of the programme for the research team to get feedback about their experiences. We anticipate that they will last about an hour and they will be audio recorded. This recording will be destroyed when we take the data from it. All your information will be kept confidential throughout and after completion of this study. We will not know until this study is completed and all data is collected whether this programme will improve the quality of life and health of those who participate.

**How many people will take part in the study?** We hope to enrol 120 people into this study. 60 people will participate in an active programme (study group) and will get support and advice to improve diet and activity. This advice will be tailored to your individual needs. The other 60 participants will receive usual care and advice (control group).

**Do I have to take part?**  It is up to you to decide whether or not to take part. If you decide to participate you will be asked to sign a consent form and given a copy to keep. If you decide to take part and change your mind later you are free to withdraw at any time without giving any reason. This will not affect the standard of care you receive.

**What are the possible benefits of taking part in this study?** Decreased physical activity and overweight have been associated with an increased risk of developing cancer again and feeling less well overall. If you partake in this research you may develop a healthier lifestyle. This may make you feel better physically and psychologically. Your cancer risk may also be reduced.

**What are the possible risks of taking part in this study?** There are no foreseeable risks involved.

**Will my information be kept confidential?** All information collected will be coded and kept confidentially in a password protected computer by the research team. If information from this study is presented or published your name and other personal information will not be used.

**What are the costs of taking part in this study?** You will not be charged for any costs associated with taking part in this study. You will not be paid to participate in this study.

**Who has reviewed and approved this study?** This study was approved by the National University of Ireland, Galway Research Ethics Committee, and by the Research Ethics Committee at Letterkenny University Hospital.

**Contact for further information:** If you have any questions concerning this study, or any problems arise please contact:

**Janice Richmond, Mary Grace Kelly,**

**Adv. Nurse Practitioner, Oncology. Clinical Research Nurse**

**Ph: 074 9123648 Ph:074 9104642**

**Email:** [**janicep.richmond@hse.ie**](mailto:janicep.richmond@hse.ie) **Email:** [**marygrace.kelly@hse.ie**](mailto:marygrace.kelly@hse.ie)

**WRITTEN CONSENT FORM**

**Study Title:** A trial to investigate the impact of a personalised self-management lifestyle programme using mobile technology on the health and wellbeing of cancer survivors.

**Principle Investigators:** Dr Janice Richmond Letterkenny University Hospital & Dr Jane Walsh, NUI Galway.

**Participant Identification No.:**

**Please initial box**

1. I confirm that I have read the information sheet for the above □

study and have had the opportunity to ask questions.

2. I am satisfied that I understand the information provided and □

have had enough time to consider the information.

3. I understand that my participation is voluntary and that I am □

free to withdraw at any time, without giving any reason, without

my legal rights being affected.

4. I agree to take part in the above study. □

Name of Participant Date Signature

Researcher Date Signature

1 copy for participant; 1 copy for researcher; 1 copy to be kept with research notes
