# Supplementary material for: Examining the Impact of an mHealth Behavior Change Intervention With a Brief In-Person Component for Cancer Survivors With Overweight or Obesity: Randomized Controlled Trial
Source: JMIR Mhealth Uhealth. 2021 Jul 5;9(7):e24915. doi: 10.2196/24915 (PMC8406099; doi:10.2196/24915)
Supplement: Multimedia Appendix 2 [file mhealth_v9i7e24915_app2.docx]

| Appendix 2. Results of 3x2 ANOVA on measures of the 6-min walk test | | | | | | | | | |
| --- | --- | --- | --- | --- | --- | --- | --- | --- | --- |
|  | | | | | | | | | |
|  | Group*Time | | | Group | | | Time | | |
|  | F  (2,242) | p | ηp2 | F  (1,121) | p | ηp2 | F  (2,242) | p | ηp2 |
| Distance Walked | 0.165 | .848 |  | 3.848 | .052 |  | 36.760 | .000 | .233 |
| Resting SBP | 1.086 | .339 |  | 1.052 | .307 |  | 10.758 | .000 | .082 |
| Resting DBP | 1.150 | .318 |  | 1.576 | .212 |  | 10.589 | .000 | .080 |
| Resting HR | 1.412 | .246 |  | .482 | .489 |  | 3.825 | .023 | .031 |
| Post-test SBP | 1.577 | .209 |  | 0.384 | .536 |  | 2.482 | .086 |  |
| Post-test DBP | 1.468 | .232 |  | 2.084 | .151 |  | 2.040 | .132 |  |
| Post-test HR | 0.770 | .464 |  | .467 | .496 |  | 2.461 | .088 |  |
| Recovery SBP | .433 | .649 |  | 2.198 | .141 |  | 16.868 | .000 | .120 |
| Recovery DBP | .431 | .650 |  | .434 | .511 |  | 5.184 | .006 | .041 |
| Recovery HR | .075 | .928 |  | .010 | .919 |  | 5.034 | .007 | .040 |
| SpO2 resting | 3.076 | .048 | .025 | 1.020 | .314 |  | .901 | .407 |  |
| SpO2 post-test | 2.743 | .066 |  | 4.986 | .027 | .040 | 1.052 | .351 |  |
| SpO2 recovery | 2.516 | .083 |  | 2.332 | .129 |  | .367 | .694 |  |
| Fatigue resting | .229 | .795 |  | .000 | .991 |  | 5.047 | .007 | .040 |
| Fatigue post-test | .121 | .886 |  | .084 | .773 |  | 3.568 | .030 | .029 |
| Fatigue recovery | .159 | .853 |  | .037 | .849 |  | 8.566 | .000 | .066 |
| Dyspnea Resting | .893 | .411 |  | .866 | .354 |  | 5.894 | .003 | .046 |
| Dyspnea post-test | 1.884 | .154 |  | 1.470 | .228 |  | 5.250 | .006 | .042 |
| Dyspnea recovery | 1.979 | .140 |  | 1.327 | .252 |  | 4.34 | .014 | .035 |
