# Supplementary material for: Examining the Impact of an mHealth Behavior Change Intervention With a Brief In-Person Component for Cancer Survivors With Overweight or Obesity: Randomized Controlled Trial
Source: JMIR Mhealth Uhealth. 2021 Jul 5;9(7):e24915. doi: 10.2196/24915 (PMC8406099; doi:10.2196/24915)
Supplement: Multimedia Appendix 3 [file mhealth_v9i7e24915_app3.docx]

| Appendix 3. Results of 3x2 ANOVA analysis on subscales of the RAND-36 Medical Outcomes Survey: Short Form | | | | | | | | | |
| --- | --- | --- | --- | --- | --- | --- | --- | --- | --- |
|  | Group*Time | | | Group | | | Time | | |
|  | F  (2,242) | p | ηp2 | F  (1,121) | p | ηp2 | F  (2,242) | p | ηp2 |
| physical functioning | .557 | .573 |  | 1.090 | .298 |  | 10.594 | .000 | .081 |
| role function– physical | .639 | .529 |  | .455 | .501 |  | 6.636 | .002 | .052 |
| role function–emotional | 1.339 | .264 |  | .955 | .330 |  | 4.824 | .009 | .038 |
| pain | .054 | .947 |  | .074 | .786 |  | 4.573 | .011 | .036 |
| emotional wellbeing | .749 | .474 |  | 2.179 | .142 |  | 12.991 | .000 | .097 |
| social functioning | .306 | .737 |  | .309 | .579 |  | 20.577 | .000 | .145 |
| energy | .797 | .452 |  | 4.230 | .042 | .034 | 27.922 | .000 | .187 |
| general health | .444 | .642 |  | .530 | .468 |  | 15.235 | .000 | .112 |
