# Supplementary material for: Examining the Impact of an mHealth Behavior Change Intervention With a Brief In-Person Component for Cancer Survivors With Overweight or Obesity: Randomized Controlled Trial
Source: JMIR Mhealth Uhealth. 2021 Jul 5;9(7):e24915. doi: 10.2196/24915 (PMC8406099; doi:10.2196/24915)
Supplement: Multimedia Appendix 4 [file mhealth_v9i7e24915_app4.docx]

| **Appendix 4.** Results of 3x2 ANOVA analysis on other Psychological Outcomes | | | | | | | | | |
| --- | --- | --- | --- | --- | --- | --- | --- | --- | --- |
|  | Group*Time | | | Group | | | Time | | |
|  | F  (2,242) | p | ηp2 | F  (1,121) | p | ηp2 | F  (2,242) | p | ηp2 |
| Loneliness | .685 | .505 |  | 6.077 | .015 | .048 | 1.514 | .222 |  |
| Fatigue (global) | 3.199 | .043 | .026 | 8.741 | .004 | .067 | 12.184 | .000 | .091 |
| Fatigue Severity | 1.517 | .221 |  | 11.033 | .001 | .084 | 5.353 | .005 | .042 |
| Fatigue Interference | 2.816 | .062 |  | 6.483 | .012 | .051 | 12.501 | .041 | .094 |
| Self-Efficacy | 2.457 | .088 |  | .270 | .605 |  | 7.192 | .001 | .056 |
| Exercise  Self-efficacy | .363 | .696 |  | 2.782 | .098 |  | 3.543 | .030 |  |
| Exercise  Social support | .078 | .925 |  | .134 | .715 |  | 2.391 | .094 |  |
