# Supplementary material for: Examining the Impact of an mHealth Behavior Change Intervention With a Brief In-Person Component for Cancer Survivors With Overweight or Obesity: Randomized Controlled Trial
Source: JMIR Mhealth Uhealth. 2021 Jul 5;9(7):e24915. doi: 10.2196/24915 (PMC8406099; doi:10.2196/24915)
Supplement: Multimedia Appendix 5 [file mhealth_v9i7e24915_app5.docx]

| **Appendix 5.** Results of 3x2 ANOVA analysis on Dietary Behavior | | | | | | | | | |
| --- | --- | --- | --- | --- | --- | --- | --- | --- | --- |
|  | Group*Time | | | Group | | | Time | | |
|  | F  (2,242) | p | ηp2 | F  (1,121) | p | ηp2 | F  (2,242) | p | ηp2 |
| Fibre | .443 | .643 |  | 3.507 | .064 |  | 1.620 | .200 |  |
| Kilocalorie | .644 | .526 |  | 2.208 | .140 |  | 12.440 | < .001 | .093 |
| Sodium | .576 | .563 |  | .859 | .356 |  | 20.292 | < .001 | .144 |
| Saturated fats | 1.067 | .346 |  | .432 | .512 |  | 15.409 | < .001 | .113 |
| Fruit | .338 | .713 |  | .697 | .405 |  | 4.589 | .011 | .037 |
| Meat | .071 | .932 |  | 0.78 | .378 |  | 8.360 | < .001 | .065 |
| Sugar | .529 | .590 |  | .221 | .639 |  | 15.941 | < .001 | .116 |
| Vegetables | .838 | .434 |  | 2.236 | .137 |  | 10.861 | < .001 | .082 |
| Alcohol | 1.249 | .289 |  | 1.293 | .258 |  | 4.759 | .009 | .038 |
| Alcoholic beverages | 2.350 | .097 |  | 1.130 | .290 |  | 5.098 | .007 | .007 |
