# Supplementary material for: Examining the Impact of an mHealth Behavior Change Intervention With a Brief In-Person Component for Cancer Survivors With Overweight or Obesity: Randomized Controlled Trial
Source: JMIR Mhealth Uhealth. 2021 Jul 5;9(7):e24915. doi: 10.2196/24915 (PMC8406099; doi:10.2196/24915)
Supplement: Multimedia Appendix 6 [file mhealth_v9i7e24915_app6.pdf]

CONSORT-EHEALTH (V 1.6.1) - Submission/Publication Form

The CONSORT-EHEALTH checklist is intended for authors of randomized trials evaluating web-based and Internet-based applications/interventions, including mobile interventions, electronic games (incl multiplayer games), social media, certain telehealth applications, and other interactive and/or networked electronic applications. Some of the items (e.g. all subitems under item 5 - description of the intervention) may also be applicable for other study designs.

The goal of the CONSORT EHEALTH checklist and guideline is to be
a) a guide for reporting for authors of RCTs,
b) to form a basis for appraisal of an ehealth trial (in terms of validity)

CONSORT-EHEALTH items/subitems are MANDATORY reporting items for studies published in the Journal of Medical Internet Research and other journals / scientific societies endorsing the checklist.

Items numbered 1., 2., 3., 4a., 4b etc are original CONSORT or CONSORT-NPT (non-pharmacologic treatment) items.
Items with Roman numerals (i., ii, iii, iv etc.) are CONSORT-EHEALTH extensions/clarifications.

As the CONSORT-EHEALTH checklist is still considered in a formative stage, we would ask that you also RATE ON A SCALE OF 1-5 how important/useful you feel each item is FOR THE PURPOSE OF THE CHECKLIST and reporting guideline (optional).

Mandatory reporting items are marked with a red *.
In the textboxes, either copy & paste the relevant sections from your manuscript into this form - please include any quotes from your manuscript in QUOTATION MARKS,
or answer directly by providing additional information not in the manuscript, or elaborating on why the item was not relevant for this study.

YOUR ANSWERS WILL BE PUBLISHED AS A SUPPLEMENTARY FILE TO YOUR PUBLICATION IN JMIR AND ARE CONSIDERED PART OF YOUR PUBLICATION (IF ACCEPTED).
Please fill in these questions diligently. Information will not be copyedited, so please use proper spelling and grammar, use correct capitalization, and avoid abbreviations.

DO NOT FORGET TO SAVE AS PDF _AND_ CLICK THE SUBMIT BUTTON SO YOUR ANSWERS ARE IN OUR DATABASE !!!

Citation Suggestion (if you append the pdf as Appendix we suggest to cite this paper in the caption):
Eysenbach G, CONSORT-EHEALTH Group
CONSORT-EHEALTH: Improving and Standardizing Evaluation Reports of Web-based and

Mobile Health Interventions
J Med Internet Res 2011;13(4):e126
URL: http://www.jmir.org/2011/4/e126/
doi: 10.2196/jmir.1923
PMID: 22209829

* Required

Your name *

First Last

Jenny Groarke

Primary Affiliation (short), City, Country *

University of Toronto, Toronto, Canada

Queen's University Belfast, Belfast, United King

Your e-mail address *

abc@gmail.com

j.groarke@qub.ac.uk

Title of your manuscript *

Provide the (draft) title of your manuscript.

Examining the Impact of a Mobile Health Behavior Change Intervention for Cancer Survivors with Overweight/Obesity: Randomized Controlled Trial

Name of your App/Software/Intervention *

If there is a short and a long/alternate name, write the short name first and add the long name in brackets.

Moving On intervention

Evaluated Version (if any)

e.g. "V1", "Release 2017-03-01", "Version 2.0.27913"

Your answer

Language(s) *

What language is the intervention/app in? If multiple languages are available, separate by comma (e.g. "English, French")

English

URL of your Intervention Website or App

e.g. a direct link to the mobile app on app in appstore (itunes, Google Play), or URL of the website. If the intervention is a DVD or hardware, you can also link to an Amazon page.

Your answer

URL of an image/screenshot (optional)

Your answer

Accessibility *

Can an enduser access the intervention presently?

- ○ access is free and open
- ○ access only for special usergroups, not open
- ○ access is open to everyone, but requires payment/subscription/in-app purchases
- ◉ app/intervention no longer accessible
- ○ Other:

Primary Medical Indication/Disease/Condition *

e.g. "Stress", "Diabetes", or define the target group in brackets after the condition, e.g. "Autism (Parents of children with)", "Alzheimers (Informal Caregivers of)"

Cancer survivors with overweight/obesity

Primary Outcomes measured in trial *

comma-separated list of primary outcomes reported in the trial

Anthropometric Measurements, Functional exe

Secondary/other outcomes

Are there any other outcomes the intervention is expected to affect?

Your answer

Recommended "Dose" *

What do the instructions for users say on how often the app should be used?

- ○ Approximately Daily
- ○ Approximately Weekly
- ○ Approximately Monthly
- ○ Approximately Yearly
- ○ "as needed"
- ◉ Other: daily self-monitoring via wearable activity monitor and weekly SMS co

Approx. Percentage of Users (starters) still using the app as recommended after 3 months *

- ◉ unknown / not evaluated
- ○ 0-10%
- ○ 11-20%
- ○ 21-30%
- ○ 31-40%
- ○ 41-50%
- ○ 51-60%
- ○ 61-70%
- ○ 71%-80%
- ○ 81-90%
- ○ 91-100%
- ○ Other:

Overall, was the app/intervention effective? *

- ○ yes: all primary outcomes were significantly better in intervention group vs control
- ◉ partly: SOME primary outcomes were significantly better in intervention group vs control
- ○ no statistically significant difference between control and intervention
- ○ potentially harmful: control was significantly better than intervention in one or more outcomes
- ○ inconclusive: more research is needed
- ○ Other:

Article Preparation Status/Stage *

At which stage in your article preparation are you currently (at the time you fill in this form)

- ○ not submitted yet - in early draft status
- ○ not submitted yet - in late draft status, just before submission
- ○ submitted to a journal but not reviewed yet
- ◉ submitted to a journal and after receiving initial reviewer comments
- ○ submitted to a journal and accepted, but not published yet
- ○ published
- ○ Other:

Journal *

If you already know where you will submit this paper (or if it is already submitted), please provide the journal name (if it is not JMIR, provide the journal name under "other")

- ( ) not submitted yet / unclear where I will submit this
- ( ) Journal of Medical Internet Research (JMIR)
- (•) JMIR mHealth and UHealth
- ( ) JMIR Serious Games
- ( ) JMIR Mental Health
- ( ) JMIR Public Health
- ( ) JMIR Formative Research
- ( ) Other JMIR sister journal
- ( ) Other:

Is this a full powered effectiveness trial or a pilot/feasibility trial? *

- ( ) Pilot/feasibility
- (•) Fully powered

Manuscript tracking number *

If this is a JMIR submission, please provide the manuscript tracking number under "other" (The ms tracking number can be found in the submission acknowledgement email, or when you login as author in JMIR. If the paper is already published in JMIR, then the ms tracking number is the four-digit number at the end of the DOI, to be found at the bottom of each published article in JMIR)

- ( ) no ms number (yet) / not (yet) submitted to / published in JMIR
- (•) Other: 24915

TITLE AND ABSTRACT

1a) TITLE: Identification as a randomized trial in the title

1a) Does your paper address CONSORT item 1a? *

I.e does the title contain the phrase "Randomized Controlled Trial"? (if not, explain the reason under "other")

- (●) yes
- ( ) Other:

1a-i) Identify the mode of delivery in the title

Identify the mode of delivery. Preferably use “web-based” and/or “mobile” and/or “electronic game” in the title. Avoid ambiguous terms like “online”, “virtual”, “interactive”. Use “Internet-based” only if Intervention includes non-web-based Internet components (e.g. email), use “computer-based” or “electronic” only if offline products are used. Use “virtual” only in the context of “virtual reality” (3-D worlds). Use “online” only in the context of “online support groups”. Complement or substitute product names with broader terms for the class of products (such as “mobile” or “smart phone” instead of “iphone”), especially if the application runs on different platforms.

	1	2	3	4	5	
subitem not at all important	○	○	○	○	○	essential

Does your paper address subitem 1a-i? *

Copy and paste relevant sections from manuscript title (include quotes in quotation marks "like this" to indicate direct quotes from your manuscript), or elaborate on this item by providing additional information not in the ms, or briefly explain why the item is not applicable/relevant for your study

Yes - "Examining the Impact of a Mobile Health Behavior Change Intervention with brief in-person component for Cancer Survivors with Overweight/Obesity: Randomized Controlled Trial"

1a-ii) Non-web-based components or important co-interventions in title

Mention non-web-based components or important co-interventions in title, if any (e.g., “with telephone support”).

	1	2	3	4	5	
subitem not at all important	○	○	○	○	○	essential

Does your paper address subitem 1a-ii?

Copy and paste relevant sections from manuscript title (include quotes in quotation marks "like this" to indicate direct quotes from your manuscript), or elaborate on this item by providing additional information not in the ms, or briefly explain why the item is not applicable/relevant for your study

Yes - "Examining the Impact of a Mobile Health Behavior Change Intervention with brief in-person component for Cancer Survivors with Overweight/Obesity: Randomized Controlled Trial"

1a-iii) Primary condition or target group in the title

Mention primary condition or target group in the title, if any (e.g., “for children with Type I Diabetes”)
Example: A Web-based and Mobile Intervention with Telephone Support for Children with Type I Diabetes: Randomized Controlled Trial

	1	2	3	4	5	
subitem not at all important	○	○	○	○	○	essential

Does your paper address subitem 1a-iii? *

Copy and paste relevant sections from manuscript title (include quotes in quotation marks "like this" to indicate direct quotes from your manuscript), or elaborate on this item by providing additional information not in the ms, or briefly explain why the item is not applicable/relevant for your study

Yes - "Examining the Impact of a Mobile Health Behavior Change Intervention with brief in-person component for Cancer Survivors with Overweight/Obesity: Randomized Controlled Trial"

1b) ABSTRACT: Structured summary of trial design, methods, results, and conclusions

NPT extension: Description of experimental treatment, comparator, care providers, centers, and blinding status.

1b-i) Key features/functionalities/components of the intervention and comparator in the METHODS section of the ABSTRACT

Mention key features/functionalities/components of the intervention and comparator in the abstract. If possible, also mention theories and principles used for designing the site. Keep in mind the needs of systematic reviewers and indexers by including important synonyms. (Note: Only report in the abstract what the main paper is reporting. If this information is missing from the main body of text, consider adding it)

	1	2	3	4	5	
subitem not at all important	○	○	○	○	○	essential

Does your paper address subitem 1b-i? *

Copy and paste relevant sections from the manuscript abstract (include quotes in quotation marks "like this" to indicate direct quotes from your manuscript), or elaborate on this item by providing additional information not in the ms, or briefly explain why the item is not applicable/relevant for your study

Yes - "Methods: A sample of 123 cancer survivors (body mass index ≥25 kg/m2) was randomly assigned to the standard care control (n=61) or intervention (n=62) condition. Group allocation was unblinded. The intervention group attended a 4-hour tailored lifestyle education and information session with physiotherapists, a dietician, and clinical psychologist to support self-management of health behavior. Over the following 12 weeks, participants engaged in personalized goal setting to incrementally increase physical activity (with feedback and review of goals through short message service text messaging contact with the research team). Direct measures of physical activity were collected using a Fitbit accelerometer. Data on anthropometric, functional exercise capacity, dietary behavior, and psychological measures were collected at face-to-face assessments in a single hospital site at baseline (T0), 12 weeks (T1; intervention end), and 24 weeks (T2; follow-up). "

1b-ii) Level of human involvement in the METHODS section of the ABSTRACT

Clarify the level of human involvement in the abstract, e.g., use phrases like “fully automated” vs. “therapist/nurse/care provider/physician-assisted” (mention number and expertise of providers involved, if any). (Note: Only report in the abstract what the main paper is reporting. If this information is missing from the main body of text, consider adding it)

	1	2	3	4	5	
subitem not at all important	○	○	○	○	○	essential

Does your paper address subitem 1b-ii?

Copy and paste relevant sections from the manuscript abstract (include quotes in quotation marks "like this" to indicate direct quotes from your manuscript), or elaborate on this item by providing additional information not in the ms, or briefly explain why the item is not applicable/relevant for your study

Yes - "Methods: A sample of 123 cancer survivors (body mass index ≥25 kg/m2) was randomly assigned to the standard care control (n=61) or intervention (n=62) condition. Group allocation was unblinded. The intervention group attended a 4-hour tailored lifestyle education and information session with physiotherapists, a dietician, and clinical psychologist to support self-management of health behavior. Over the following 12 weeks, participants engaged in personalized goal setting to incrementally increase physical activity (with feedback and review of goals through short message service text messaging contact with the research team). Direct measures of physical activity were collected using a Fitbit accelerometer. Data on anthropometric, functional exercise capacity, dietary behavior, and psychological measures were collected at face-to-face assessments in a single hospital site at baseline (T0), 12 weeks (T1; intervention end), and 24 weeks (T2; follow-up). "

1b-iii) Open vs. closed, web-based (self-assessment) vs. face-to-face assessments in the METHODS section of the ABSTRACT

Mention how participants were recruited (online vs. offline), e.g., from an open access website or from a clinic or a closed online user group (closed usergroup trial), and clarify if this was a purely web-based trial, or there were face-to-face components (as part of the intervention or for assessment). Clearly say if outcomes were self-assessed through questionnaires (as common in web-based trials). Note: In traditional offline trials, an open trial (open-label trial) is a type of clinical trial in which both the researchers and participants know which treatment is being administered. To avoid confusion, use “blinded” or “unblinded” to indicated the level of blinding instead of “open”, as “open” in web-based trials usually refers to “open access” (i.e. participants can self-enrol). (Note: Only report in the abstract what the main paper is reporting. If this information is missing from the main body of text, consider adding it)

	1	2	3	4	5	
subitem not at all important	○	○	○	○	○	essential

Does your paper address subitem 1b-iii?

Copy and paste relevant sections from the manuscript abstract (include quotes in quotation marks "like this" to indicate direct quotes from your manuscript), or elaborate on this item by providing additional information not in the ms, or briefly explain why the item is not applicable/relevant for your study

Yes - "Methods: A sample of 123 cancer survivors (body mass index ≥25 kg/m2) was randomly assigned to the standard care control (n=61) or intervention (n=62) condition. Group allocation was unblinded. The intervention group attended a 4-hour tailored lifestyle education and information session with physiotherapists, a dietician, and clinical psychologist to support self-management of health behavior. Over the following 12 weeks, participants engaged in personalized goal setting to incrementally increase physical activity (with feedback and review of goals through short message service text messaging contact with the research team). Direct measures of physical activity were collected using a Fitbit accelerometer. Data on anthropometric, functional exercise capacity, dietary behavior, and psychological measures were collected at face-to-face assessments in a single hospital site at baseline (T0), 12 weeks (T1; intervention end), and 24 weeks (T2; follow-up). "

1b-iv) RESULTS section in abstract must contain use data

Report number of participants enrolled/assessed in each group, the use/uptake of the intervention (e.g., attrition/adherence metrics, use over time, number of logins etc.), in addition to primary/secondary outcomes. (Note: Only report in the abstract what the main paper is reporting. If this information is missing from the main body of text, consider adding it)

	1	2	3	4	5	
subitem not at all important	○	○	○	○	○	essential

Does your paper address subitem 1b-iv?

Copy and paste relevant sections from the manuscript abstract (include quotes in quotation marks "like this" to indicate direct quotes from your manuscript), or elaborate on this item by providing additional information not in the ms, or briefly explain why the item is not applicable/relevant for your study

Yes - "Results: Rates of attrition were 21% for the control condition and 14% for the intervention condition. Using intent-to-treat analysis significant reductions in body mass index (BMI) ($F(2,242) = 4.149$, $p = .017$, $\eta p2= .033$) and waist circumference ($F(2,242) = 3.342$, $p = .037$, $np2 = .027$) were seen in the intervention group. Over the 24-week study BMI was reduced by 0.52 in the intervention condition, relative to a non-significant reduction of 0.11 in the control arm. Waist circumference reduced by 3.02cm in the intervention relative to 1.82cm in the control condition. Physical activity level was significantly higher in the intervention group on 8 of the 12 weeks of the intervention phase, and on 5 of the 12 weeks of the follow-up period, accounting for up to 2500 additional steps per day ($M = 2032$, $SD = 270$). "

1b-v) CONCLUSIONS/DISCUSSION in abstract for negative trials

Conclusions/Discussions in abstract for negative trials: Discuss the primary outcome - if the trial is negative (primary outcome not changed), and the intervention was not used, discuss whether negative results are attributable to lack of uptake and discuss reasons. (Note: Only report in the abstract what the main paper is reporting. If this information is missing from the main body of text, consider adding it)

	1	2	3	4	5	
subitem not at all important	○	○	○	○	○	essential

Does your paper address subitem 1b-v?

Copy and paste relevant sections from the manuscript abstract (include quotes in quotation marks "like this" to indicate direct quotes from your manuscript), or elaborate on this item by providing additional information not in the ms, or briefly explain why the item is not applicable/relevant for your study

Yes - "Conclusions: The results demonstrate that for cancer survivors with a BMI ≥ 25 lifestyle education and personalized goal setting using mobile technology can yield significant change on clinically relevant health indicators. Further research is needed to elucidate to mechanisms for behavior change and explore the capacity for mHealth interventions to improve broader health and wellbeing outcomes in the growing population of cancer survivors."

INTRODUCTION

2a) In INTRODUCTION: Scientific background and explanation of rationale

2a-i) Problem and the type of system/solution

Describe the problem and the type of system/solution that is object of the study: intended as stand-alone intervention vs. incorporated in broader health care program? Intended for a particular patient population? Goals of the intervention, e.g., being more cost-effective to other interventions, replace or complement other solutions? (Note: Details about the intervention are provided in “Methods” under 5)

	1	2	3	4	5	
subitem not at all important	○	○	○	○	○	essential

Does your paper address subitem 2a-i? *

Copy and paste relevant sections from the manuscript (include quotes in quotation marks "like this" to indicate direct quotes from your manuscript), or elaborate on this item by providing additional information not in the ms, or briefly explain why the item is not applicable/relevant for your study

Yes - "There is an average of 35,000 new cases of cancer diagnosed each year in Ireland, representing a doubling of cases in the last 25 years [1]. At the same time, cancer survivorship in Ireland is also increasing, with survival at five years from diagnosis having increased from 42% in 1994 to 62% in 2019, with cancer survivors now making up 4% of the Irish population [1].

There is consistent evidence of a positive association between overweight, obesity and all-cause morbidity and mortality [2]. High body mass index (BMI), poor diet, and lack of physical activity are identifiable risk factors for cancer development, and in cancer survivors, these factors can increase the risk of a secondary cancer or a subsequent primary cancer [3,4]. Cancer and its' treatment can result in fatigue, physical inactivity, and loss of muscular strength [5]. Approximately 50% of cancer survivors are overweight [6], and research has linked obesity to a 46% increased risk of developing distant metastases in women [7]. Cognizant of the consequences of morbidity and mortality, there is a need to facilitate rehabilitation of cancer survivors to reduce BMI, improve diet and increase physical activity. "

2a-ii) Scientific background, rationale: What is known about the (type of) system

Scientific background, rationale: What is known about the (type of) system that is the object of the study (be sure to discuss the use of similar systems for other conditions/diagnoses, if appropiate), motivation for the study, i.e. what are the reasons for and what is the context for this specific study, from which stakeholder viewpoint is the study performed, potential impact of findings [2]. Briefly justify the choice of the comparator.

	1	2	3	4	5	
subitem not at all important	○	○	○	○	○	essential

Does your paper address subitem 2a-ii? *

Copy and paste relevant sections from the manuscript (include quotes in quotation marks "like this" to indicate direct quotes from your manuscript), or elaborate on this item by providing additional information not in the ms, or briefly explain why the item is not applicable/relevant for your study

Yes - "mHealth interventions incorporating relevant BCTs have the potential to improve health and wellbeing outcomes. However, there are a limited number of mHealth interventions with cancer survivors that describe content in terms of BCTs. A recent systematic review identified 15 digital health behavior change interventions for cancer survivors, concluding that digital interventions may improve physical activity and reduce BMI, but that findings regarding dietary behavior and wellbeing outcomes are mixed [23]. Although many of the included studies were pilot or feasibility trials, they highlight the potential for mHealth interventions to improve health behavior and health outcomes among cancer survivors. All but one study included in this review relied on self-report measures of physical activity [24]. Self-report measures have known limitations, such as, recall bias, misinterpretation of items, and overestimation of activity relative to direct measures, such as, accelerometer devices [25–28]. Relative to accelerometers, error rates of between 35-79% have been observed on self-report measures [29,30]. Further, it is noteworthy that only two of the included digital interventions purposively sampled cancer survivors with overweight/obesity [31,32]. This population is arguably most in need of intervention, and health behavior change interventions using non-digital modes of delivery can improve outcomes for this cohort of survivors [33,34]. Overall, there is a need for more large scale randomized controlled trials to provide high quality evidence regarding the impact of mHealth interventions on objectively measured outcomes with cancer survivors with overweight/obesity. "

2b) In INTRODUCTION: Specific objectives or hypotheses

Does your paper address CONSORT subitem 2b? *

Copy and paste relevant sections from the manuscript (include quotes in quotation marks "like this" to indicate direct quotes from your manuscript), or elaborate on this item by providing additional information not in the ms, or briefly explain why the item is not applicable/relevant for your study

Yes - "The aim of this study was to investigate the impact of a personalized mHealth (mobile technology) behavior change intervention on physical and psychological health outcomes for a group of cancer survivors with overweight/obesity. More specifically, this project examined the impact of lifestyle education and personalized goal setting, compared with standard medical care, on physical activity (step count), as well as other behavioral, clinical and psychological outcomes."

METHODS

3a) Description of trial design (such as parallel, factorial) including allocation ratio

Does your paper address CONSORT subitem 3a? *

Copy and paste relevant sections from the manuscript (include quotes in quotation marks "like this" to indicate direct quotes from your manuscript), or elaborate on this item by providing additional information not in the ms, or briefly explain why the item is not applicable/relevant for your study

Yes "A 2-arm, parallel, open-label RCT design was used to investigate the impact of the intervention versus standard care on clinical, psychological and health behavior outcomes. "

3b) Important changes to methods after trial commencement (such as eligibility criteria), with reasons

Does your paper address CONSORT subitem 3b? *

Copy and paste relevant sections from the manuscript (include quotes in quotation marks "like this" to indicate direct quotes from your manuscript), or elaborate on this item by providing additional information not in the ms, or briefly explain why the item is not applicable/relevant for your study

Yes - throughout methods section

3b-i) Bug fixes, Downtimes, Content Changes

Bug fixes, Downtimes, Content Changes: ehealth systems are often dynamic systems. A description of changes to methods therefore also includes important changes made on the intervention or comparator during the trial (e.g., major bug fixes or changes in the functionality or content) (5-iii) and other “unexpected events” that may have influenced study design such as staff changes, system failures/downtimes, etc. [2].

	1	2	3	4	5	
subitem not at all important	○	○	○	○	○	essential

Does your paper address subitem 3b-i?

Copy and paste relevant sections from the manuscript (include quotes in quotation marks "like this" to indicate direct quotes from your manuscript), or elaborate on this item by providing additional information not in the ms, or briefly explain why the item is not applicable/relevant for your study

Yes - "Within two weeks of receipt a number of participants reported challenges using their Fitbit. As a result, all participants were invited to attend one of two technical support sessions. Twelve attended a session and received hands-on support and troubleshooting advice regarding their device from the research team (JG and MGK). Following the implementation of EU General Data Protection Regulation (May 2018) participants were automatically logged out of their Fitbit app. However, this was possible to fix at a distance over the phone or via text message. "

4a) Eligibility criteria for participants

Does your paper address CONSORT subitem 4a? *

Copy and paste relevant sections from the manuscript (include quotes in quotation marks "like this" to indicate direct quotes from your manuscript), or elaborate on this item by providing additional information not in the ms, or briefly explain why the item is not applicable/relevant for your study

Yes - detailed in methods section

4a-i) Computer / Internet literacy

Computer / Internet literacy is often an implicit “de facto” eligibility criterion - this should be explicitly clarified.

	1	2	3	4	5	
subitem not at all important	○	○	○	○	○	essential

Does your paper address subitem 4a-i?

Copy and paste relevant sections from the manuscript (include quotes in quotation marks "like this" to indicate direct quotes from your manuscript), or elaborate on this item by providing additional information not in the ms, or briefly explain why the item is not applicable/relevant for your study

Yes - "Adults aged 18 to 70 years, having a calculated BMI equal to or greater than 25 kg/m2, with a solid cancer and who had completed active cancer treatment (those continuing on endocrine therapy were permitted inclusion), attended Oncology Services in Letterkenny University Hospital during the recruitment phase (December 2017-January 2018), and were willing to use mobile technology were eligible to participate. " ....
"A total of 10 eligible participants who were willing to use mobile technology but did not own a smartphone were provided with an Amazon Fire 7 Tablet."

4a-ii) Open vs. closed, web-based vs. face-to-face assessments:

Open vs. closed, web-based vs. face-to-face assessments: Mention how participants were recruited (online vs. offline), e.g., from an open access website or from a clinic, and clarify if this was a purely web-based trial, or there were face-to-face components (as part of the intervention or for assessment), i.e., to what degree got the study team to know the participant. In online-only trials, clarify if participants were quasi-anonymous and whether having multiple identities was possible or whether technical or logistical measures (e.g., cookies, email confirmation, phone calls) were used to detect/prevent these.

	1	2	3	4	5	
subitem not at all important	○	○	○	○	○	essential

Does your paper address subitem 4a-ii? *

Copy and paste relevant sections from the manuscript (include quotes in quotation marks "like this" to indicate direct quotes from your manuscript), or elaborate on this item by providing additional information not in the ms, or briefly explain why the item is not applicable/relevant for your study

Yes - "Participants were recruited from Oncology Services in Letterkenny University Hospital. One hundred and fifty-nine eligible participants (18-70 years, BMI ≥ 25, active cancer treatment completed) were identified sequentially from the oncology outpatient waiting list (N = 347) by the clinical team. The clinical team contacted these participants by telephone and described the aims and design of the study and asked if they were willing to use mobile technology. Prospective participants who expressed interested in the study were sent a participant information sheet and consent form (see Appendix 1). Informed written consent was provided by 123 participants (77.3% response rate) who all then attended in-person baseline assessments. "

4a-iii) Information giving during recruitment

Information given during recruitment. Specify how participants were briefed for recruitment and in the informed consent procedures (e.g., publish the informed consent documentation as appendix, see also item X26), as this information may have an effect on user self-selection, user expectation and may also bias results.

	1	2	3	4	5	
subitem not at all important	○	○	○	○	○	essential

Does your paper address subitem 4a-iii?

Copy and paste relevant sections from the manuscript (include quotes in quotation marks "like this" to indicate direct quotes from your manuscript), or elaborate on this item by providing additional information not in the ms, or briefly explain why the item is not applicable/relevant for your study

Yes - " Prospective participants who expressed interested in the study were sent a participant information sheet and consent form (see Appendix 1)"

4b) Settings and locations where the data were collected

Does your paper address CONSORT subitem 4b? *

Copy and paste relevant sections from the manuscript (include quotes in quotation marks "like this" to indicate direct quotes from your manuscript), or elaborate on this item by providing additional information not in the ms, or briefly explain why the item is not applicable/relevant for your study

Yes - throughout methods section

4b-i) Report if outcomes were (self-)assessed through online questionnaires

Clearly report if outcomes were (self-)assessed through online questionnaires (as common in web-based trials) or otherwise.

	1	2	3	4	5	
subitem not at all important	○	○	○	○	○	essential

Does your paper address subitem 4b-i? *

Copy and paste relevant sections from the manuscript (include quotes in quotation marks "like this" to indicate direct quotes from your manuscript), or elaborate on this item by providing additional information not in the ms, or briefly explain why the item is not applicable/relevant for your study

Yes - "The study was not blinded but step count, one of the main outcome measures was recorded directly using the Fitbit device. " ...

4b-ii) Report how institutional affiliations are displayed

Report how institutional affiliations are displayed to potential participants [on ehealth media], as affiliations with prestigious hospitals or universities may affect volunteer rates, use, and reactions with regards to an intervention.(Not a required item – describe only if this may bias results)

	1	2	3	4	5	
subitem not at all important	○	○	○	○	○	essential

Does your paper address subitem 4b-ii?

Copy and paste relevant sections from the manuscript (include quotes in quotation marks "like this" to indicate direct quotes from your manuscript), or elaborate on this item by providing additional information not in the ms, or briefly explain why the item is not applicable/relevant for your study

Yes - "Prospective participants who expressed interested in the study were sent a participant information sheet and consent form (see Appendix 1)"

5) The interventions for each group with sufficient details to allow replication, including how and when they were actually administered

5-i) Mention names, credential, affiliations of the developers, sponsors, and owners

Mention names, credential, affiliations of the developers, sponsors, and owners [6] (if authors/evaluators are owners or developer of the software, this needs to be declared in a “Conflict of interest” section or mentioned elsewhere in the manuscript).

	1	2	3	4	5	
subitem not at all important	○	○	○	○	○	essential

Does your paper address subitem 5-i?

Copy and paste relevant sections from the manuscript (include quotes in quotation marks "like this" to indicate direct quotes from your manuscript), or elaborate on this item by providing additional information not in the ms, or briefly explain why the item is not applicable/relevant for your study

Yes - detailed sections describing intervention and control condition.

5-ii) Describe the history/development process

Describe the history/development process of the application and previous formative evaluations (e.g., focus groups, usability testing), as these will have an impact on adoption/use rates and help with interpreting results.

	1	2	3	4	5	
subitem not at all important	○	○	○	○	○	essential

Does your paper address subitem 5-ii?

Copy and paste relevant sections from the manuscript (include quotes in quotation marks "like this" to indicate direct quotes from your manuscript), or elaborate on this item by providing additional information not in the ms, or briefly explain why the item is not applicable/relevant for your study

Yes - detailed section on intervention development, links to protocol paper describing in more detail. "This complex intervention was delivered through mHealth technology and included BCTs which aimed to improve clinical, psychological and health behavior outcomes. The full details are described in the study protocol [35]. In summary, the intervention had two components: "

5-iii) Revisions and updating

Revisions and updating. Clearly mention the date and/or version number of the application/intervention (and comparator, if applicable) evaluated, or describe whether the intervention underwent major changes during the evaluation process, or whether the development and/or content was “frozen” during the trial. Describe dynamic components such as news feeds or changing content which may have an impact on the replicability of the intervention (for unexpected events see item 3b).

	1	2	3	4	5	
subitem not at all important	○	○	○	○	○	essential

Does your paper address subitem 5-iii?

Copy and paste relevant sections from the manuscript (include quotes in quotation marks "like this" to indicate direct quotes from your manuscript), or elaborate on this item by providing additional information not in the ms, or briefly explain why the item is not applicable/relevant for your study

No - not applicable, though discussion section makes some recommendation for future iterations.

5-iv) Quality assurance methods

Provide information on quality assurance methods to ensure accuracy and quality of information provided [1], if applicable.

	1	2	3	4	5	
subitem not at all important	○	○	○	○	○	essential

Does your paper address subitem 5-iv?

Copy and paste relevant sections from the manuscript (include quotes in quotation marks "like this" to indicate direct quotes from your manuscript), or elaborate on this item by providing additional information not in the ms, or briefly explain why the item is not applicable/relevant for your study

Yes - "To maximize power and conform to intent-to-treat analysis, missing data were handled using the expectation-maximization (EM) algorithm. A non-significant Little's MCAR test showed the data was missing completely at random (X2 = 113.29, df = 30080, p = 1.00), therefore data substitution methods were deemed appropriate. For step count data specifically, EM data substitution was applied only for the 107 participants who received a Fitbit. The 16 participants in the intent-to-treat group (i.e., those who could not attend the initial session where Fitbits were distributed) are not included in the missing value analysis and EM data substitution for the analyses of group differences in step count. For those participants included in the missing value analysis of step count, days with zero steps recorded treated as missing data, self-reported adherence to Fitbit wear was not recorded. "

5-v) Ensure replicability by publishing the source code, and/or providing screenshots/screen-capture video, and/or providing flowcharts of the algorithms used

Ensure replicability by publishing the source code, and/or providing screenshots/screen-capture video, and/or providing flowcharts of the algorithms used. Replicability (i.e., other researchers should in principle be able to replicate the study) is a hallmark of scientific reporting.

	1	2	3	4	5	
subitem not at all important	○	○	○	○	○	essential

Does your paper address subitem 5-v?

Copy and paste relevant sections from the manuscript (include quotes in quotation marks "like this" to indicate direct quotes from your manuscript), or elaborate on this item by providing additional information not in the ms, or briefly explain why the item is not applicable/relevant for your study

Yes - sufficient detail regarding the intervention is provided to ensure reproducibility.

5-vi) Digital preservation

Digital preservation: Provide the URL of the application, but as the intervention is likely to change or disappear over the course of the years; also make sure the intervention is archived (Internet Archive, webcitation.org, and/or publishing the source code or screenshots/videos alongside the article). As pages behind login screens cannot be archived, consider creating demo pages which are accessible without login.

	1	2	3	4	5	
subitem not at all important	○	○	○	○	○	essential

Does your paper address subitem 5-vi?

Copy and paste relevant sections from the manuscript (include quotes in quotation marks "like this" to indicate direct quotes from your manuscript), or elaborate on this item by providing additional information not in the ms, or briefly explain why the item is not applicable/relevant for your study

N/A - the intervention involves the use of commercially available mobile technology (SMS and Fitbit) in combination with behaviour change techniques

5-vii) Access

Access: Describe how participants accessed the application, in what setting/context, if they had to pay (or were paid) or not, whether they had to be a member of specific group. If known, describe how participants obtained “access to the platform and Internet” [1]. To ensure access for editors/reviewers/readers, consider to provide a “backdoor” login account or demo mode for reviewers/readers to explore the application (also important for archiving purposes, see vi).

	1	2	3	4	5	
subitem not at all important	○	○	○	○	○	essential

Does your paper address subitem 5-vii? *

Copy and paste relevant sections from the manuscript (include quotes in quotation marks "like this" to indicate direct quotes from your manuscript), or elaborate on this item by providing additional information not in the ms, or briefly explain why the item is not applicable/relevant for your study

Yes - participants were provided with a Fitbit tracker, that was paired with their mobile device. If they did not have a mobile device they were provided with an Amazon Fire tablet.

5-viii) Mode of delivery, features/functionalities/components of the intervention and comparator, and the theoretical framework

Describe mode of delivery, features/functionalities/components of the intervention and comparator, and the theoretical framework [6] used to design them (instructional strategy [1], behaviour change techniques, persuasive features, etc., see e.g., [7, 8] for terminology). This includes an in-depth description of the content (including where it is coming from and who developed it) [1],” whether [and how] it is tailored to individual circumstances and allows users to track their progress and receive feedback” [6]. This also includes a description of communication delivery channels and – if computer-mediated communication is a component – whether communication was synchronous or asynchronous [6]. It also includes information on presentation strategies [1], including page design principles, average amount of text on pages, presence of hyperlinks to other resources, etc. [1].

	1	2	3	4	5	
subitem not at all important	○	○	○	○	○	essential

Does your paper address subitem 5-viii? *

Copy and paste relevant sections from the manuscript (include quotes in quotation marks "like this" to indicate direct quotes from your manuscript), or elaborate on this item by providing additional information not in the ms, or briefly explain why the item is not applicable/relevant for your study

Yes - "In summary, the intervention had two components:
1. A 4-hour lifestyle education and information session (Week 1) delivered by healthcare professionals (three physiotherapists, one dietician, one clinical psychologist). Physiotherapists demonstrated a series of daily strengthening exercises and recommended schedules of moderate intensity physical activity. The dietician delivered a comprehensive overview on healthy eating, answered numerous questions which clarified misinformation on nutrition, and specifically advised participants to reduce their caloric intake, reduce red meat, processed meat, salt and sugar, and increase fruit, vegetable, and fiber intake. The clinical psychologist offered practical strategies for problem solving, identifying barriers to change, and preventing relapse. The BCTs included in this session and the corresponding code from the BCT Taxonomy V1 [16], were goal setting (outcome) (1.3), provide information on consequences of behavior to the individual (5.1), demonstration of the behavior (6.1), provide instruction on how to perform the behavior (4.1), problem solving (1.2), goal setting (behavior) (1.1), and action planning (1.4). These BCTs applied to both physical activity and dietary behavior change. The mode of delivery for this component of the intervention was face-to-face human contact in real time to groups of participants. During this session all participants in attendance were provided with a Fitbit Alta.
2. An 8-week goal setting intervention (Weeks 4-12) delivered using mobile technology (i.e., Fitbit Alta accelerometer plus short message service (SMS) contact). Participants received weekly text messages with feedback on their average daily step count, and a goal to increase their step count by 10% in the coming week. The BCTs included in the personalized goal setting intervention were self-monitoring of behavior (2.3), feedback on behavior (2.2), goal setting (behavior) (1.1), graded tasks (8.7), social reward (10.4), and review behavior goal(s) (1.5). The mode of delivery for this intervention component was human contact at a distance using nonautomated SMS text messages facilitated using digital wearable technology (i.e., Fitbit Alta). Participants continued to wear the Fitbit for the remainder of the study (24 weeks), but the personalized goal setting intervention ceased at 12 weeks".

5-ix) Describe use parameters

Describe use parameters (e.g., intended “doses” and optimal timing for use). Clarify what instructions or recommendations were given to the user, e.g., regarding timing, frequency, heaviness of use, if any, or was the intervention used ad libitum.

	1	2	3	4	5	
subitem not at all important	○	○	○	○	○	essential

Does your paper address subitem 5-ix?

Copy and paste relevant sections from the manuscript (include quotes in quotation marks "like this" to indicate direct quotes from your manuscript), or elaborate on this item by providing additional information not in the ms, or briefly explain why the item is not applicable/relevant for your study

Yes - "In summary, the intervention had two components:
1. A 4-hour lifestyle education and information session (Week 1) delivered by healthcare professionals (three physiotherapists, one dietician, one clinical psychologist). Physiotherapists demonstrated a series of daily strengthening exercises and recommended schedules of moderate intensity physical activity. The dietician delivered a comprehensive overview on healthy eating, answered numerous questions which clarified misinformation on nutrition, and specifically advised participants to reduce their caloric intake, reduce red meat, processed meat, salt and sugar, and increase fruit, vegetable, and fiber intake. The clinical psychologist offered practical strategies for problem solving, identifying barriers to change, and preventing relapse. The BCTs included in this session and the corresponding code from the BCT Taxonomy V1 [16], were goal setting (outcome) (1.3), provide information on consequences of behavior to the individual (5.1), demonstration of the behavior (6.1), provide instruction on how to perform the behavior (4.1), problem solving (1.2), goal setting (behavior) (1.1), and action planning (1.4). These BCTs applied to both physical activity and dietary behavior change. The mode of delivery for this component of the intervention was face-to-face human contact in real time to groups of participants. During this session all participants in attendance were provided with a Fitbit Alta.
2. An 8-week goal setting intervention (Weeks 4-12) delivered using mobile technology (i.e., Fitbit Alta accelerometer plus short message service (SMS) contact). Participants received weekly text messages with feedback on their average daily step count, and a goal to increase their step count by 10% in the coming week. The BCTs included in the personalized goal setting intervention were self-monitoring of behavior (2.3), feedback on behavior (2.2), goal setting (behavior) (1.1), graded tasks (8.7), social reward (10.4), and review behavior goal(s) (1.5). The mode of delivery for this intervention component was human contact at a distance using nonautomated SMS text messages facilitated using digital wearable technology (i.e., Fitbit Alta). Participants continued to wear the Fitbit for the remainder of the study (24 weeks), but the personalized goal setting intervention ceased at 12 weeks."

5-x) Clarify the level of human involvement

Clarify the level of human involvement (care providers or health professionals, also technical assistance) in the e-intervention or as co-intervention (detail number and expertise of professionals involved, if any, as well as “type of assistance offered, the timing and frequency of the support, how it is initiated, and the medium by which the assistance is delivered”. It may be necessary to distinguish between the level of human involvement required for the trial, and the level of human involvement required for a routine application outside of a RCT setting (discuss under item 21 – generalizability).

	1	2	3	4	5	
subitem not at all important	○	○	○	○	○	essential

Does your paper address subitem 5-x?

Copy and paste relevant sections from the manuscript (include quotes in quotation marks "like this" to indicate direct quotes from your manuscript), or elaborate on this item by providing additional information not in the ms, or briefly explain why the item is not applicable/relevant for your study

Yes - "In summary, the intervention had two components:

1. A 4-hour lifestyle education and information session (Week 1) delivered by healthcare professionals (three physiotherapists, one dietician, one clinical psychologist). Physiotherapists demonstrated a series of daily strengthening exercises and recommended schedules of moderate intensity physical activity. The dietician delivered a comprehensive overview on healthy eating, answered numerous questions which clarified misinformation on nutrition, and specifically advised participants to reduce their caloric intake, reduce red meat, processed meat, salt and sugar, and increase fruit, vegetable, and fiber intake. The clinical psychologist offered practical strategies for problem solving, identifying barriers to change, and preventing relapse. The BCTs included in this session and the corresponding code from the BCT Taxonomy V1 [16], were goal setting (outcome) (1.3), provide information on consequences of behavior to the individual (5.1), demonstration of the behavior (6.1), provide instruction on how to perform the behavior (4.1), problem solving (1.2), goal setting (behavior) (1.1), and action planning (1.4). These BCTs applied to both physical activity and dietary behavior change. The mode of delivery for this component of the intervention was face-to-face human contact in real time to groups of participants. During this session all participants in attendance were provided with a Fitbit Alta.
2. An 8-week goal setting intervention (Weeks 4-12) delivered using mobile technology (i.e., Fitbit Alta accelerometer plus short message service (SMS) contact). Participants received weekly text messages with feedback on their average daily step count, and a goal to increase their step count by 10% in the coming week. The BCTs included in the personalized goal setting intervention were self-monitoring of behavior (2.3), feedback on behavior (2.2), goal setting (behavior) (1.1), graded tasks (8.7), social reward (10.4), and review behavior goal(s) (1.5). The mode of delivery for this intervention component was human contact at a distance using nonautomated SMS text messages facilitated using digital wearable technology (i.e., Fitbit Alta). Participants continued to wear the Fitbit for the remainder of the study (24 weeks), but the personalized goal setting intervention ceased at 12 weeks."

5-xi) Report any prompts/reminders used

Report any prompts/reminders used: Clarify if there were prompts (letters, emails, phone calls, SMS) to use the application, what triggered them, frequency etc. It may be necessary to distinguish between the level of prompts/reminders required for the trial, and the level of prompts/reminders for a routine application outside of a RCT setting (discuss under item 21 – generalizability).

	1	2	3	4	5	
subitem not at all important	○	○	○	○	○	essential

Does your paper address subitem 5-xi? *

Copy and paste relevant sections from the manuscript (include quotes in quotation marks "like this" to indicate direct quotes from your manuscript), or elaborate on this item by providing additional information not in the ms, or briefly explain why the item is not applicable/relevant for your study

Yes - participants were contacted weekly regarding their step count "To facilitate the goal setting intervention a weighted average for daily step count was calculated for participants with at least 5 observations in a week. Participants who showed no activity for more than 2 days a week were contacted to verify no technical issues. There were a number of possible reasons for someone to have 0 steps on a given day (eg., the participant didn't wear the monitor, the Fitbit failed to record). These reasons were not recorded, and self-reported adherence to monitor wear was not measured"

5-xii) Describe any co-interventions (incl. training/support)

Describe any co-interventions (incl. training/support): Clearly state any interventions that are provided in addition to the targeted eHealth intervention, as ehealth intervention may not be designed as stand-alone intervention. This includes training sessions and support [1]. It may be necessary to distinguish between the level of training required for the trial, and the level of training for a routine application outside of a RCT setting (discuss under item 21 – generalizability.

	1	2	3	4	5	
subitem not at all important	○	○	○	○	○	essential

Does your paper address subitem 5-xii? *

Copy and paste relevant sections from the manuscript (include quotes in quotation marks "like this" to indicate direct quotes from your manuscript), or elaborate on this item by providing additional information not in the ms, or briefly explain why the item is not applicable/relevant for your study

Yes - "To facilitate the goal setting intervention a weighted average for daily step count was calculated for participants with at least 5 observations in a week. Participants who showed no activity for more than 2 days a week were contacted to verify no technical issues. There were a number of possible reasons for someone to have 0 steps on a given day (eg., the participant didn't wear the monitor, the Fitbit failed to record). These reasons were not recorded, and self-reported adherence to monitor wear was not measured. Within two weeks of receipt a number of participants reported challenges using their Fitbit. As a result, all participants were invited to attend one of two technical support sessions. Twelve attended a session and received hands-on support and troubleshooting advice regarding their device from the research team (JG and MGK)"

6a) Completely defined pre-specified primary and secondary outcome measures, including how and when they were assessed

Does your paper address CONSORT subitem 6a? *

Copy and paste relevant sections from the manuscript (include quotes in quotation marks "like this" to indicate direct quotes from your manuscript), or elaborate on this item by providing additional information not in the ms, or briefly explain why the item is not applicable/relevant for your study

Yes - described in detail under materials section. outcomes were assessed through direct observation and self-report measures in the clinical setting, and using direct measure for step count (i.e., fitbit).

6a-i) Online questionnaires: describe if they were validated for online use and apply CHERRIES items to describe how the questionnaires were designed/deployed

If outcomes were obtained through online questionnaires, describe if they were validated for online use and apply CHERRIES items to describe how the questionnaires were designed/deployed [9].

	1	2	3	4	5	
subitem not at all important	○	○	○	○	○	essential

Does your paper address subitem 6a-i?

Copy and paste relevant sections from manuscript text

Yes - "self-reported adherence to monitor wear was not measured"

6a-ii) Describe whether and how “use” (including intensity of use/dosage) was defined/measured/monitored

Describe whether and how “use” (including intensity of use/dosage) was defined/measured/monitored (logins, logfile analysis, etc.). Use/adoption metrics are important process outcomes that should be reported in any ehealth trial.

	1	2	3	4	5	
subitem not at all important	○	○	○	○	○	essential

Does your paper address subitem 6a-ii?

Copy and paste relevant sections from manuscript text

Your answer

6a-iii) Describe whether, how, and when qualitative feedback from participants was obtained

Describe whether, how, and when qualitative feedback from participants was obtained (e.g., through emails, feedback forms, interviews, focus groups).

	1	2	3	4	5	
subitem not at all important	○	○	○	○	○	essential

Does your paper address subitem 6a-iii?

Copy and paste relevant sections from manuscript text

Yes - qualitative interviews were carried out retrsopectively and reported in a separate publication (Groarke et al., 2021), cited throughout current RCT paper.

6b) Any changes to trial outcomes after the trial commenced, with reasons

Does your paper address CONSORT subitem 6b? *

Copy and paste relevant sections from the manuscript (include quotes in quotation marks "like this" to indicate direct quotes from your manuscript), or elaborate on this item by providing additional information not in the ms, or briefly explain why the item is not applicable/relevant for your study

Yes - no changes were implemented, as per published protocol.

7a) How sample size was determined

NPT: When applicable, details of whether and how the clustering by care provides or centers was addressed

7a-i) Describe whether and how expected attrition was taken into account when calculating the sample size

Describe whether and how expected attrition was taken into account when calculating the sample size.

	1	2	3	4	5	
subitem not at all important	○	○	○	○	○	essential

Does your paper address subitem 7a-i?

Copy and paste relevant sections from manuscript title (include quotes in quotation marks "like this" to indicate direct quotes from your manuscript), or elaborate on this item by providing additional information not in the ms, or briefly explain why the item is not applicable/relevant for your study

Yes - "The statistical program G*Power was used to conduct power analysis. With two groups (intervention and control), 3 measurements (baseline, time 1, and time 2), an assumed correlation among repeated measures of 0.3, and a small-medium effect size and a power of 0.8, the recommended sample size for repeated measures analysis of variance (ANOVA) was 102. A final sample size of 123 was calculated based on an attrition rate of 20% observed in similar studies using mobile technology interventions with cancer survivors [37]. "

7b) When applicable, explanation of any interim analyses and stopping guidelines

Does your paper address CONSORT subitem 7b? *

Copy and paste relevant sections from the manuscript (include quotes in quotation marks "like this" to indicate direct quotes from your manuscript), or elaborate on this item by providing additional information not in the ms, or briefly explain why the item is not applicable/relevant for your study

Not reported - no interim analyses were conducted

8a) Method used to generate the random allocation sequence

NPT: When applicable, how care providers were allocated to each trial group

Does your paper address CONSORT subitem 8a? *

Copy and paste relevant sections from the manuscript (include quotes in quotation marks "like this" to indicate direct quotes from your manuscript), or elaborate on this item by providing additional information not in the ms, or briefly explain why the item is not applicable/relevant for your study

Yes - "Participants were randomized to either the intervention or the standard care control condition using a computerized random number generator (enrollment was carried out by MGK and JR, randomization and group allocation was carried out by JG)".

8b) Type of randomisation; details of any restriction (such as blocking and block size)

Does your paper address CONSORT subitem 8b? *

Copy and paste relevant sections from the manuscript (include quotes in quotation marks "like this" to indicate direct quotes from your manuscript), or elaborate on this item by providing additional information not in the ms, or briefly explain why the item is not applicable/relevant for your study

Yes - "Participants were randomized to either the intervention or the standard care control condition using a computerized random number generator (enrollment was carried out by MGK and JR, randomization and group allocation was carried out by JG). "

9) Mechanism used to implement the random allocation sequence (such as sequentially numbered containers), describing any steps taken to conceal the sequence until interventions were assigned

Does your paper address CONSORT subitem 9? *

Copy and paste relevant sections from the manuscript (include quotes in quotation marks "like this" to indicate direct quotes from your manuscript), or elaborate on this item by providing additional information not in the ms, or briefly explain why the item is not applicable/relevant for your study

Yes - "Participants were randomized to either the intervention or the standard care control condition using a computerized random number generator (enrollment was carried out by MGK and JR, randomization and group allocation was carried out by JG). "

10) Who generated the random allocation sequence, who enrolled participants, and who assigned participants to interventions

Does your paper address CONSORT subitem 10? *

Copy and paste relevant sections from the manuscript (include quotes in quotation marks "like this" to indicate direct quotes from your manuscript), or elaborate on this item by providing additional information not in the ms, or briefly explain why the item is not applicable/relevant for your study

Yes - "Participants were randomized to either the intervention or the standard care control condition using a computerized random number generator (enrollment was carried out by MGK and JR, randomization and group allocation was carried out by JG). "

11a) If done, who was blinded after assignment to interventions (for example, participants, care providers, those assessing outcomes) and how

NPT: Whether or not administering co-interventions were blinded to group assignment

11a-i) Specify who was blinded, and who wasn't

Specify who was blinded, and who wasn't. Usually, in web-based trials it is not possible to blind the participants [1, 3] (this should be clearly acknowledged), but it may be possible to blind outcome assessors, those doing data analysis or those administering co-interventions (if any).

	1	2	3	4	5	
subitem not at all important	○	○	○	○	○	essential

Does your paper address subitem 11a-i? *

Copy and paste relevant sections from the manuscript (include quotes in quotation marks "like this" to indicate direct quotes from your manuscript), or elaborate on this item by providing additional information not in the ms, or briefly explain why the item is not applicable/relevant for your study

Yes - "The study was not blinded but step count, one of the main outcome measures was recorded directly using the Fitbit device. " and abstract "Group allocation was unblinded. "

11a-ii) Discuss e.g., whether participants knew which intervention was the "intervention of interest" and which one was the "comparator"

Informed consent procedures (4a-ii) can create biases and certain expectations - discuss e.g., whether participants knew which intervention was the "intervention of interest" and which one was the "comparator".

	1	2	3	4	5	
subitem not at all important	○	○	○	○	○	essential

Does your paper address subitem 11a-ii?

Copy and paste relevant sections from the manuscript (include quotes in quotation marks "like this" to indicate direct quotes from your manuscript), or elaborate on this item by providing additional information not in the ms, or briefly explain why the item is not applicable/relevant for your study

Yes - "The study was not blinded but step count, one of the main outcome measures was recorded directly using the Fitbit device. " and abstract "Group allocation was unblinded. "

11b) If relevant, description of the similarity of interventions

(this item is usually not relevant for ehealth trials as it refers to similarity of a placebo or sham intervention to a active medication/intervention)

Does your paper address CONSORT subitem 11b? *

Copy and paste relevant sections from the manuscript (include quotes in quotation marks "like this" to indicate direct quotes from your manuscript), or elaborate on this item by providing additional information not in the ms, or briefly explain why the item is not applicable/relevant for your study

Yes - extensively throughout method and direct comparison offered in Table 1.

12a) Statistical methods used to compare groups for primary and secondary outcomes

NPT: When applicable, details of whether and how the clustering by care providers or centers was addressed

Does your paper address CONSORT subitem 12a? *

Copy and paste relevant sections from the manuscript (include quotes in quotation marks "like this" to indicate direct quotes from your manuscript), or elaborate on this item by providing additional information not in the ms, or briefly explain why the item is not applicable/relevant for your study

Yes - section in methods section detailing this.

12a-i) Imputation techniques to deal with attrition / missing values

Imputation techniques to deal with attrition / missing values: Not all participants will use the intervention/comparator as intended and attrition is typically high in ehealth trials. Specify how participants who did not use the application or dropped out from the trial were treated in the statistical analysis (a complete case analysis is strongly discouraged, and simple imputation techniques such as LOCF may also be problematic [4]).

	1	2	3	4	5	
subitem not at all important	○	○	○	○	○	essential

Does your paper address subitem 12a-i? *

Copy and paste relevant sections from the manuscript (include quotes in quotation marks "like this" to indicate direct quotes from your manuscript), or elaborate on this item by providing additional information not in the ms, or briefly explain why the item is not applicable/relevant for your study

Yes - "To maximize power and conform to intent-to-treat analysis, missing data were handled using the expectation-maximization (EM) algorithm. A non-significant Little's MCAR test showed the data was missing completely at random (X2 = 113.29, df = 30080, p = 1.00), therefore data substitution methods were deemed appropriate. For step count data specifically, EM data substitution was applied only for the 107 participants who received a Fitbit. The 16 participants in the intent-to-treat group (i.e., those who could not attend the initial session where Fitbits were distributed) are not included in the missing value analysis and EM data substitution for the analyses of group differences in step count. "

12b) Methods for additional analyses, such as subgroup analyses and adjusted analyses

Does your paper address CONSORT subitem 12b? *

Copy and paste relevant sections from the manuscript (include quotes in quotation marks "like this" to indicate direct quotes from your manuscript), or elaborate on this item by providing additional information not in the ms, or briefly explain why the item is not applicable/relevant for your study

Yes - "A series of 3 (time: baseline [T0], 12 weeks [T1], and 24 weeks [T2]) × 2 (group: control and intervention) mixed ANOVAs were performed to determine the effect of the intervention on clinical, psychological, and health behavior outcomes. In the case of a significant interaction effect follow up independent sample t-tests were conducted to investigate between group differences at each timepoint and one-way ANOVAs were conducted to identify within group differences across timepoints. Independent samples t-tests were used to analyze group differences (control and intervention) in average daily step count across the 24 weeks of the study. "

X26) REB/IRB Approval and Ethical Considerations [recommended as subheading under "Methods"] (not a CONSORT item)

X26-i) Comment on ethics committee approval

	1	2	3	4	5	
subitem not at all important	○	○	○	○	○	essential

Does your paper address subitem X26-i?

Copy and paste relevant sections from the manuscript (include quotes in quotation marks "like this" to indicate direct quotes from your manuscript), or elaborate on this item by providing additional information not in the ms, or briefly explain why the item is not applicable/relevant for your study

Yes - "The design of this study was approved by the National University of Ireland, Galway Research Ethics Committee on September 12, 2017 (Ref: 17/MAY/20), and by the Research Ethics Committee at Letterkenny University Hospital on May 2, 2017. "

x26-ii) Outline informed consent procedures

Outline informed consent procedures e.g., if consent was obtained offline or online (how? Checkbox, etc.?), and what information was provided (see 4a-ii). See [6] for some items to be included in informed consent documents.

	1	2	3	4	5	
subitem not at all important	○	○	○	○	○	essential

Does your paper address subitem X26-ii?

Copy and paste relevant sections from the manuscript (include quotes in quotation marks "like this" to indicate direct quotes from your manuscript), or elaborate on this item by providing additional information not in the ms, or briefly explain why the item is not applicable/relevant for your study

Yes - "Prospective participants who expressed interested in the study were sent a participant information sheet and consent form (see Appendix 1). Informed written consent was provided by 123 participants (77.3% response rate) who all then attended in-person baseline assessments."

X26-iii) Safety and security procedures

Safety and security procedures, incl. privacy considerations, and any steps taken to reduce the likelihood or detection of harm (e.g., education and training, availability of a hotline)

	1	2	3	4	5	
subitem not at all important	○	○	○	○	○	essential

Does your paper address subitem X26-iii?

Copy and paste relevant sections from the manuscript (include quotes in quotation marks "like this" to indicate direct quotes from your manuscript), or elaborate on this item by providing additional information not in the ms, or briefly explain why the item is not applicable/relevant for your study

Yes - the participant information sheet included as an appendix details the complaints procedure.

RESULTS

13a) For each group, the numbers of participants who were randomly assigned, received intended treatment, and were analysed for the primary outcome

NPT: The number of care providers or centers performing the intervention in each group and the number of patients treated by each care provider in each center

Does your paper address CONSORT subitem 13a? *

Copy and paste relevant sections from the manuscript (include quotes in quotation marks "like this" to indicate direct quotes from your manuscript), or elaborate on this item by providing additional information not in the ms, or briefly explain why the item is not applicable/relevant for your study

Yes - see Figure 1 participant flow chart.

13b) For each group, losses and exclusions after randomisation, together with reasons

Does your paper address CONSORT subitem 13b? (NOTE: Preferably, this is shown in a CONSORT flow diagram) *

Copy and paste relevant sections from the manuscript (include quotes in quotation marks "like this" to indicate direct quotes from your manuscript), or elaborate on this item by providing additional information not in the ms, or briefly explain why the item is not applicable/relevant for your study

Yes - Flow diagram is presented as Figure 1, with attrition in each arm. Reasons for declining to participate are presented in methods "Of the 36 eligible participants who did not consent to participate (22.7%), 28 were not interested, 3 were waiting for surgery, 1 had chronic obstructive pulmonary disease, 1 was undergoing recurrence workup, 2 had young children, and 1 did not drive (see Figure 1)."

13b-i) Attrition diagram

Strongly recommended: An attrition diagram (e.g., proportion of participants still logging in or using the intervention/comparator in each group plotted over time, similar to a survival curve) or other figures or tables demonstrating usage/dose/engagement.

	1	2	3	4	5	
subitem not at all important	○	○	○	○	○	essential

Does your paper address subitem 13b-i?

Copy and paste relevant sections from the manuscript or cite the figure number if applicable (include quotes in quotation marks "like this" to indicate direct quotes from your manuscript), or elaborate on this item by providing additional information not in the ms, or briefly explain why the item is not applicable/relevant for your study

Yes - Flow diagram is presented as Figure 1, with attrition in each arm. Reasons for declining to participate are presented in methods "Of the 36 eligible participants who did not consent to participate (22.7%), 28 were not interested, 3 were waiting for surgery, 1 had chronic obstructive pulmonary disease, 1 was undergoing recurrence workup, 2 had young children, and 1 did not drive (see Figure 1)."

14a) Dates defining the periods of recruitment and follow-up

Does your paper address CONSORT subitem 14a? *

Copy and paste relevant sections from the manuscript (include quotes in quotation marks "like this" to indicate direct quotes from your manuscript), or elaborate on this item by providing additional information not in the ms, or briefly explain why the item is not applicable/relevant for your study

specific dates of enrollment and assessments are provided in the published protocol and trial registration, the fixes section described introduction of GDPR regulation

14a-i) Indicate if critical “secular events” fell into the study period

Indicate if critical “secular events” fell into the study period, e.g., significant changes in Internet resources available or “changes in computer hardware or Internet delivery resources”

	1	2	3	4	5	
subitem not at all important	○	○	○	○	○	essential

Does your paper address subitem 14a-i?

Copy and paste relevant sections from the manuscript (include quotes in quotation marks "like this" to indicate direct quotes from your manuscript), or elaborate on this item by providing additional information not in the ms, or briefly explain why the item is not applicable/relevant for your study

specific dates of enrolment and assessments are provided in the published protocol and trial registration, the fixes section described introduction of GDPR regulation

14b) Why the trial ended or was stopped (early)

Does your paper address CONSORT subitem 14b? *

Copy and paste relevant sections from the manuscript (include quotes in quotation marks "like this" to indicate direct quotes from your manuscript), or elaborate on this item by providing additional information not in the ms, or briefly explain why the item is not applicable/relevant for your study

No - not applicable to the current trial, fully completed.

15) A table showing baseline demographic and clinical characteristics for each group

NPT: When applicable, a description of care providers (case volume, qualification, expertise, etc.) and centers (volume) in each group

Does your paper address CONSORT subitem 15? *

Copy and paste relevant sections from the manuscript (include quotes in quotation marks "like this" to indicate direct quotes from your manuscript), or elaborate on this item by providing additional information not in the ms, or briefly explain why the item is not applicable/relevant for your study

Yes - detailed in Table 2.

15-i) Report demographics associated with digital divide issues

In ehealth trials it is particularly important to report demographics associated with digital divide issues, such as age, education, gender, social-economic status, computer/Internet/ehealth literacy of the participants, if known.

	1	2	3	4	5	
subitem not at all important	○	○	○	○	○	essential

Does your paper address subitem 15-i? *

Copy and paste relevant sections from the manuscript (include quotes in quotation marks "like this" to indicate direct quotes from your manuscript), or elaborate on this item by providing additional information not in the ms, or briefly explain why the item is not applicable/relevant for your study

Yes - demographic data on age is provided. inclusion criteria were 'willingness to use technology' and the impact of the digital divide is discussed in limitations section "Furthermore, inclusion criteria required that participants had a willingness to use mobile technology. This may have contributed to a digital divide, in the sense that prospective participants who may have benefited from the intervention were excluded because they were inexperienced or uncomfortable using digital technologies. Is it worth noting that no-one was excluded for lack of mobile technology, participants who did not have access to but were willing to engage with mobile technology were provided with a mobile device (Amazon Fire tablet) by the research team. Finally, participants in the trial had access to technical support from the research team as needed".

16) For each group, number of participants (denominator) included in each analysis and whether the analysis was by original assigned groups

16-i) Report multiple “denominators” and provide definitions

Report multiple “denominators” and provide definitions: Report N’s (and effect sizes) “across a range of study participation [and use] thresholds” [1], e.g., N exposed, N consented, N used more than x times, N used more than y weeks, N participants “used” the intervention/comparator at specific pre-defined time points of interest (in absolute and relative numbers per group). Always clearly define “use” of the intervention.

	1	2	3	4	5	
subitem not at all important	○	○	○	○	○	essential

Does your paper address subitem 16-i? *

Copy and paste relevant sections from the manuscript (include quotes in quotation marks "like this" to indicate direct quotes from your manuscript), or elaborate on this item by providing additional information not in the ms, or briefly explain why the item is not applicable/relevant for your study

Yes - reported in Figure 1, intent-to-treat analysis is described in missing data section.

16-ii) Primary analysis should be intent-to-treat

Primary analysis should be intent-to-treat, secondary analyses could include comparing only “users”, with the appropriate caveats that this is no longer a randomized sample (see 18-i).

	1	2	3	4	5	
subitem not at all important	○	○	○	○	○	essential

Does your paper address subitem 16-ii?

Copy and paste relevant sections from the manuscript (include quotes in quotation marks "like this" to indicate direct quotes from your manuscript), or elaborate on this item by providing additional information not in the ms, or briefly explain why the item is not applicable/relevant for your study

Yes - reported in Figure 1, intent-to-treat analysis is described in missing data section.

17a) For each primary and secondary outcome, results for each group, and the estimated effect size and its precision (such as 95% confidence interval)

Does your paper address CONSORT subitem 17a? *

Copy and paste relevant sections from the manuscript (include quotes in quotation marks "like this" to indicate direct quotes from your manuscript), or elaborate on this item by providing additional information not in the ms, or briefly explain why the item is not applicable/relevant for your study

Yes - effect sizes are provided for statistical analyses

17a-i) Presentation of process outcomes such as metrics of use and intensity of use

In addition to primary/secondary (clinical) outcomes, the presentation of process outcomes such as metrics of use and intensity of use (dose, exposure) and their operational definitions is critical. This does not only refer to metrics of attrition (13-b) (often a binary variable), but also to more continuous exposure metrics such as "average session length". These must be accompanied by a technical description how a metric like a "session" is defined (e.g., timeout after idle time) [1] (report under item 6a).

	1	2	3	4	5	
subitem not at all important	○	○	○	○	○	essential

Does your paper address subitem 17a-i?

Copy and paste relevant sections from the manuscript (include quotes in quotation marks "like this" to indicate direct quotes from your manuscript), or elaborate on this item by providing additional information not in the ms, or briefly explain why the item is not applicable/relevant for your study

No - metrics around frequency of app usage or 'engagement' were not measured in the current study.

17b) For binary outcomes, presentation of both absolute and relative effect sizes is recommended

Does your paper address CONSORT subitem 17b? *

Copy and paste relevant sections from the manuscript (include quotes in quotation marks "like this" to indicate direct quotes from your manuscript), or elaborate on this item by providing additional information not in the ms, or briefly explain why the item is not applicable/relevant for your study

Although none of the outcomes assessed were binary - throughout results mean difference and effect sizes are reported.

18) Results of any other analyses performed, including subgroup analyses and adjusted analyses, distinguishing pre-specified from exploratory

Does your paper address CONSORT subitem 18? *

Copy and paste relevant sections from the manuscript (include quotes in quotation marks "like this" to indicate direct quotes from your manuscript), or elaborate on this item by providing additional information not in the ms, or briefly explain why the item is not applicable/relevant for your study

yes - all analyses are intention-to-treat i.e., not subgroup analyses.

18-i) Subgroup analysis of comparing only users

A subgroup analysis of comparing only users is not uncommon in ehealth trials, but if done, it must be stressed that this is a self-selected sample and no longer an unbiased sample from a randomized trial (see 16-iii).

	1	2	3	4	5	
subitem not at all important	○	○	○	○	○	essential

Does your paper address subitem 18-i?

Copy and paste relevant sections from the manuscript (include quotes in quotation marks "like this" to indicate direct quotes from your manuscript), or elaborate on this item by providing additional information not in the ms, or briefly explain why the item is not applicable/relevant for your study

yes - all analyses are intention-to-treat i.e., not subgroup analyses.

19) All important harms or unintended effects in each group

(for specific guidance see CONSORT for harms)

Does your paper address CONSORT subitem 19? *

Copy and paste relevant sections from the manuscript (include quotes in quotation marks "like this" to indicate direct quotes from your manuscript), or elaborate on this item by providing additional information not in the ms, or briefly explain why the item is not applicable/relevant for your study

Yes and No - potential privacy breaches were discussed, and qualitative data were collected and reported separately.

19-i) Include privacy breaches, technical problems

Include privacy breaches, technical problems. This does not only include physical “harm” to participants, but also incidents such as perceived or real privacy breaches [1], technical problems, and other unexpected/unintended incidents. “Unintended effects” also includes unintended positive effects [2].

	1	2	3	4	5	
subitem not at all important	○	○	○	○	○	essential

Does your paper address subitem 19-i?

Copy and paste relevant sections from the manuscript (include quotes in quotation marks "like this" to indicate direct quotes from your manuscript), or elaborate on this item by providing additional information not in the ms, or briefly explain why the item is not applicable/relevant for your study

No - Fixes are discussed in relation to GDPR and data protection

19-ii) Include qualitative feedback from participants or observations from staff/researchers

Include qualitative feedback from participants or observations from staff/researchers, if available, on strengths and shortcomings of the application, especially if they point to unintended/unexpected effects or uses. This includes (if available) reasons for why people did or did not use the application as intended by the developers.

	1	2	3	4	5	
subitem not at all important	○	○	○	○	○	essential

Does your paper address subitem 19-ii?

Copy and paste relevant sections from the manuscript (include quotes in quotation marks "like this" to indicate direct quotes from your manuscript), or elaborate on this item by providing additional information not in the ms, or briefly explain why the item is not applicable/relevant for your study

No - qualitative interviews were conducted and are reported in a separate paper. "Groarke JM, Richmond J, Sharry JM, et al. Acceptability of a Mobile Health Behavior Change Intervention for Cancer Survivors With Obesity or Overweight: Nested Mixed Methods Study Within a Randomized Controlled Trial. JMIR MHealth UHealth. 2021;9(2):e18288. doi:10.2196/18288"

DISCUSSION

22) Interpretation consistent with results, balancing benefits and harms, and considering other relevant evidence

NPT: In addition, take into account the choice of the comparator, lack of or partial blinding, and unequal expertise of care providers or centers in each group

22-i) Restate study questions and summarize the answers suggested by the data, starting with primary outcomes and process outcomes (use)

Restate study questions and summarize the answers suggested by the data, starting with primary outcomes and process outcomes (use).

	1	2	3	4	5	
subitem not at all important	○	○	○	○	○	essential

Does your paper address subitem 22-i? *

Copy and paste relevant sections from the manuscript (include quotes in quotation marks "like this" to indicate direct quotes from your manuscript), or elaborate on this item by providing additional information not in the ms, or briefly explain why the item is not applicable/relevant for your study

Yes - "Principal findings
The aim of this trial was to examine the impact of a personalized mobile health behavior change intervention on clinical, psychological and health behavior outcomes among a group of cancer survivors with overweight/obesity. The results show that the intervention yielded several significant benefits over and above that shown in the standard care control group. The intervention group had a significantly greater reduction in BMI than those in the control group. This reduction in BMI was maintained at the 24-week follow-up. Relative to the control group, there was a significantly greater reduction in waist circumference in the intervention group. At follow-up, there was a modest reduction in BMI (0.52) and waist circumference (3.02cm) with small to medium effect sizes. In relation to behavioral outcomes, participants in the intervention group had significantly higher physical activity during both the intervention phase (8 out of the 12 weeks) and the follow-up phase (5 out of the 12 weeks) compared with those in the control group. Participants in the intervention were averaging approximately 2,000 extra steps per day (the equivalent of one mile or 20 minutes of physical activity [46]). There was however no significant change in functional exercise capacity, dietary behavior, or psychological outcomes. "

22-ii) Highlight unanswered new questions, suggest future research

Highlight unanswered new questions, suggest future research.

	1	2	3	4	5	
subitem not at all important	○	○	○	○	○	essential

Does your paper address subitem 22-ii?

Copy and paste relevant sections from the manuscript (include quotes in quotation marks "like this" to indicate direct quotes from your manuscript), or elaborate on this item by providing additional information not in the ms, or briefly explain why the item is not applicable/relevant for your study

yes - "The success of the goal setting intervention for increasing step count in the current study is encouraging, and future digital interventions should consider goal setting in relation to dietary behavior in combination with physical activity goals. "

20) Trial limitations, addressing sources of potential bias, imprecision, and, if relevant, multiplicity of analyses

20-i) Typical limitations in ehealth trials

Typical limitations in ehealth trials: Participants in ehealth trials are rarely blinded. Ehealth trials often look at a multiplicity of outcomes, increasing risk for a Type I error. Discuss biases due to non-use of the intervention/usability issues, biases through informed consent procedures, unexpected events.

	1	2	3	4	5	
subitem not at all important	○	○	○	○	○	essential

Does your paper address subitem 20-i? *

Copy and paste relevant sections from the manuscript (include quotes in quotation marks "like this" to indicate direct quotes from your manuscript), or elaborate on this item by providing additional information not in the ms, or briefly explain why the item is not applicable/relevant for your study

Yes - "Participants were randomized to conditions to reduce selection bias, and the use of intention-to-treat analysis limited the impact of attrition bias. However, it was not possible to blind participants or outcome assessors in the current study, a limitation common to many digital health interventions [36]. This may have introduced performance and/or detection bias, and therefore results should be interpreted with caution. "

21) Generalisability (external validity, applicability) of the trial findings

NPT: External validity of the trial findings according to the intervention, comparators, patients, and care providers or centers involved in the trial

21-i) Generalizability to other populations

Generalizability to other populations: In particular, discuss generalizability to a general Internet population, outside of a RCT setting, and general patient population, including applicability of the study results for other organizations

	1	2	3	4	5	
subitem not at all important	○	○	○	○	○	essential

Does your paper address subitem 21-i?

Copy and paste relevant sections from the manuscript (include quotes in quotation marks "like this" to indicate direct quotes from your manuscript), or elaborate on this item by providing additional information not in the ms, or briefly explain why the item is not applicable/relevant for your study

Yes - "The participants were mostly female breast cancer survivors. This is representative of trends in the wider literature [60] but may limit the generalizability of the findings to people with other types of cancer, and to people of other genders in the wider population. Furthermore, inclusion criteria required that participants had a willingness to use mobile technology. This may have contributed to a digital divide, in the sense that prospective participants who may have benefited from the intervention were excluded because they were inexperienced or uncomfortable using digital technologies. Is it worth noting that no-one was excluded for lack of mobile technology, participants who did not have access to but were willing to engage with mobile technology were provided with a mobile device (Amazon Fire tablet) by the research team."

21-ii) Discuss if there were elements in the RCT that would be different in a routine application setting

Discuss if there were elements in the RCT that would be different in a routine application setting (e.g., prompts/reminders, more human involvement, training sessions or other co-interventions) and what impact the omission of these elements could have on use, adoption, or outcomes if the intervention is applied outside of a RCT setting.

	1	2	3	4	5	
subitem not at all important	○	○	○	○	○	essential

Does your paper address subitem 21-ii?

Copy and paste relevant sections from the manuscript (include quotes in quotation marks "like this" to indicate direct quotes from your manuscript), or elaborate on this item by providing additional information not in the ms, or briefly explain why the item is not applicable/relevant for your study

Yes - "Finally, participants in the trial had access to technical support from the research team as needed. This was front loaded as support was needed more frequently at commencement when participants were becoming familiar with the technology. This may not be feasible in standard oncology care, limiting the applicability of the findings outside of a RCT setting. A custom-built front-end software was used to bulk export participants' step count data to facilitate the weekly goal setting intervention, as such, something similar may be needed if applying the same intervention in a healthcare setting (e.g., [61]). Future research will be needed to identify potential implementation issues delivering this intervention in clinical settings.".

OTHER INFORMATION

23) Registration number and name of trial registry

Does your paper address CONSORT subitem 23? *

Copy and paste relevant sections from the manuscript (include quotes in quotation marks "like this" to indicate direct quotes from your manuscript), or elaborate on this item by providing additional information not in the ms, or briefly explain why the item is not applicable/relevant for your study

Yes - "Trial Registration: ISRCTN-18676721 https://doi.org/10.1186/ISRCTN18676721"

24) Where the full trial protocol can be accessed, if available

Does your paper address CONSORT subitem 24? *

Cite a Multimedia Appendix, other reference, or copy and paste relevant sections from the manuscript (include quotes in quotation marks "like this" to indicate direct quotes from your manuscript), or elaborate on this item by providing additional information not in the ms, or briefly explain why the item is not applicable/relevant for your study

Yes - "International Registered Report Identifier (IRRID): DERR1-10.2196/13214"

25) Sources of funding and other support (such as supply of drugs), role of funders

Does your paper address CONSORT subitem 25? *

Copy and paste relevant sections from the manuscript (include quotes in quotation marks "like this" to indicate direct quotes from your manuscript), or elaborate on this item by providing additional information not in the ms, or briefly explain why the item is not applicable/relevant for your study

Yes - "Funding
This research was funded by a grant awarded to JR and JW by the Irish Cancer Society with support from Relay for Life Donegal. "

X27) Conflicts of Interest (not a CONSORT item)

X27-i) State the relation of the study team towards the system being evaluated

In addition to the usual declaration of interests (financial or otherwise), also state the relation of the study team towards the system being evaluated, i.e., state if the authors/evaluators are distinct from or identical with the developers/sponsors of the intervention.

	1	2	3	4	5	
subitem not at all important	○	○	○	○	○	essential

Does your paper address subitem X27-i?

Copy and paste relevant sections from the manuscript (include quotes in quotation marks "like this" to indicate direct quotes from your manuscript), or elaborate on this item by providing additional information not in the ms, or briefly explain why the item is not applicable/relevant for your study

Yes - "Conflicts of Interest
None declared"

About the CONSORT EHEALTH checklist

As a result of using this checklist, did you make changes in your manuscript? *

- ( ) yes, major changes
- (•) yes, minor changes
- ( ) no

What were the most important changes you made as a result of using this checklist?

Your answer

How much time did you spend on going through the checklist INCLUDING making changes in your manuscript *

It was time consuming - roughly 3 additional hours.

As a result of using this checklist, do you think your manuscript has improved? *

- (x) yes
- ( ) no
- ( ) Other:

Would you like to become involved in the CONSORT EHEALTH group?

This would involve for example becoming involved in participating in a workshop and writing an "Explanation and Elaboration" document

- ( ) yes
- (x) no
- ( ) Other:

Clear selection

Any other comments or questions on CONSORT EHEALTH

Your answer

STOP - Save this form as PDF before you click submit

To generate a record that you filled in this form, we recommend to generate a PDF of this page (on a Mac, simply select "print" and then select "print as PDF") before you submit it.

When you submit your (revised) paper to JMIR, please upload the PDF as supplementary file.

Don't worry if some text in the textboxes is cut off, as we still have the complete information in our database. Thank you!

Final step: Click submit !

Click submit so we have your answers in our database!

Submit

Never submit passwords through Google Forms.

This content is neither created nor endorsed by Google. Report Abuse - Terms of Service - Privacy Policy

Google Forms
